# Deconstructing empirical fitness seascapes across scales of granularity

**DOI:** 10.1093/g3journal/jkag166

**Published:** 2026-06-26

**Authors:** Swathi Nachiar Manivannan, C Brandon Ogbunugafor

**Affiliations:** Department of Ecology and Evolutionary Biology,Yale University, 165 Prospect Street, New Haven, CT 06511, United States; Department of Ecology and Evolutionary Biology,Yale University, 165 Prospect Street, New Haven, CT 06511, United States; Santa Fe Institute, 1399 Hyde Park Road, Santa Fe, NM 87501, United States

**Keywords:** fitness landscapes, fitness seascapes, reaction norm, environmental epistasis, adaptive trajectories, locus robustness, landscape topography, PEQG2026

## Abstract

The fitness landscape metaphor remains resonant in evolutionary theory and has facilitated the birth of newer concepts, like the fitness seascape, that consider the role of environmental context in shaping the dynamics of evolution. Since its emergence, the seascape has appeared in numerous studies examining how different and fluctuating environments shape evolutionary outcomes. Despite growing interest, we lack comprehensive examinations of how environmental context shapes features of fitness seascapes. In this study, we address this gap by deconstructing empirical fitness seascapes across scales of granularity: loci, locus interactions (epistasis), alleles, trajectories, and entire seascapes. For each, we examine how environmental context influences qualitative and quantitative aspects of seascapes, and find that they change appreciably, with patterns specific to individual systems of study. We also quantify how much each scale varies across environments, and find that certain scales tend to be more sensitive to context than others. In summary, we reflect on the implications of the seascape metaphor for the incorporation of environmental effects into theoretical population genetics, for understanding how the environment shapes evolution in disease systems, and for contemporary bioengineering efforts.

## Teaser

This study examines fitness seascapes, maps of how genetic mutations affect an organism's success while accounting for environmental context. Manivannan and Ogbunugafor analyzed 12 published datasets, mostly from drug-resistant microbes and parasites, breaking each down across five scales: individual mutations, mutation interactions, whole genotypes, evolutionary pathways, and overall landscape shape. At every scale, environment reshaped the genotype-phenotype relationship, but patterns were specific to each biological system rather than universal. Notably, environment strongly influenced genotype performance and which evolutionary routes were accessible, while mutation interactions stayed relatively stable. These findings show why environmental context matters for predicting and steering evolution.

## Introduction

The fitness or adaptive landscape is a central metaphor in evolutionary theory that maps genotypes (or combinations of alleles) to fitness values. It has been an integral part of evolutionary genetics since its formal introduction by Sewall Wright in 1932 (Wright 1932), and has spawned a subfield focused on how the shape (or topography) of fitness landscapes dictates the dynamics of evolution at the molecular scale (Tenaillon et al. 1999; Gavrilets 2004; Kryazhimskiy et al. 2009; De Visser and Krug 2014; Hartl 2014; Ascensao and Desai 2025). It has also helped to develop detailed pictures of phenomena such as adaptive trajectories, epistasis, evolvability, and various other forces operating on molecular evolution (Smith et al. 2002; Watson et al. 2011; Pitzer and Affenzeller 2012; Kryazhimskiy et al. 2014; Fragata et al. 2019). We note that the term “fitness landscape” is used to describe two distinct classes of models: *genotype-to-fitness* landscapes, in which discrete genotypes are mapped directly to fitness values, and *phenotype-to-fitness* landscapes, in which genotypes are first mapped to a continuous phenotype that then determines fitness (e.g. Fisher’s geometric model Fisher 1930, Lande’s model Lande 1976). In this study, we focus exclusively on genotype-to-fitness landscapes and their environmental extensions (genotype-to-fitness seascapes), in which fitness is measured empirically for each genotype in each environment. The strength of the fitness landscape metaphor lies in its relative simplicity: because of the high-dimensionality of genotype-phenotype space, most canonical studies of fitness landscapes have focused on static environments.

However, for many years, a body of work has emerged that examines the case where the fitness landscape topography changes across environments. The ambition here is to transform the study of fitness landscapes into a more realistic instrument, as the living world can be safely described as a highly capricious, multi-environment setting. This notion was first formalized by David Merrell as the “adaptive seascape” (or fitness seascape), an iteration of the fitness landscape that considers how environmental context shapes evolution at the molecular level (Merrell 1994). Ville Mustonen and Michael Lässig then introduced a formal framework for the study of adaptation as a nonequilibrium phenomenon in fluctuating environments (Mustonen and Lässig 2009) to reflect the biological reality of adaptive evolution as well as in the context of anthropogenically driven environmental changes (Bank 2022). This has culminated in a literature that has explored how different environmental contexts craft adaptive trajectories and landscape topographies to answer questions about adaptive evolution (Agur and Slobodkin 1986; Grefenstette 1999; Cvijović et al. 2015; Ogbunugafor et al. 2016; Saakian et al. 2019; Trubenova et al. 2019; Cairns et al. 2022; Chen J et al. 2022), niche construction (Han and Hui 2014; Tanaka et al. 2020), and speciation (Cheetham 1993; Gavrilets 2010). However, evolution in dynamic environmental contexts remains relatively understudied, which has amplified the need for a better understanding of how contextual changes can drive adaptive evolution (Carja et al. 2014; Lässig et al. 2017; Agarwala and Fisher 2019; Kumawat et al. 2025).

The richness of the fitness landscape metaphor resides in the number of different questions one can ask of it. For example, previous studies have used empirical fitness landscapes to examine questions as disparate as the distribution of fitness effects (Martin and Lenormand 2006; Martin et al. 2007) to speciation (De Visser et al. 2009). This breadth of inquiry is possible because fitness landscapes are intrinsically composed of different kinds of biological information, which are relevant to different areas of evolutionary and population genetics. Studies have used fitness landscapes to ask questions about mutational steps (Rozen et al. 2008; Wagner 2023), plasticity (Peacor et al. 2006), epistasis (Martin et al. 2007; Weinreich et al. 2013; Bank 2022), and landscape topography (Szendro et al. 2013). In this study, we propose a framework for computing how changing environmental contexts influence different quantitative features of seascapes across scales of granularity. Specifically, we examine the manner in which the environmental context influences mutation effects, mutation interactions (epistasis), alleles, trajectories, and descriptions of entire landscapes, such as ruggedness. In doing so, we attempt to deconstruct the anatomy of molecular evolution.

Our framework comes at a time when evolutionary biologists are acquiring more and more empirical data and beginning to uncover the intricacies of how genetic information maps onto phenotypic traits and the fitness of organisms. We first discuss how our framework separately analyzes different scales of the fitness landscape (from individual loci to whole-landscape topography) and how each scale captures aspects of the capacity of populations to generate adaptive variation (evolvability), before testing our framework on an assortment of empirical fitness seascapes. Finally, we communicate the implications of our findings and the relevance of our framework to broader discussions in evolutionary genetics and evolvability.

## Materials and methods

A majority of the empirical fitness landscapes used in this study were consolidated and analyzed in a 2018 study (Weinreich et al. 2018). Our dataset selection was guided by the following criteria: we included published, combinatorially complete fitness landscapes that were measured across at least two distinct environments or along an environmental gradient, for which raw fitness data were publicly available. We began with the datasets compiled by Weinreich et al. (2018) and supplemented these with additional seascapes that met these criteria, namely, two on dihydrofolate reductase (DHFR) (Rodrigues et al. 2016; Guerrero et al. 2019; Ogbunugafor 2022), and one on ${\text{bla}}_{TEM}$ (a *β*-lactamase) cefotaxime resistance in different bacterial species (Kosterlitz et al. 2023). We note that other fitness seascape datasets exist in the literature (e.g. King et al. 2025); however, these were either published after our analysis was largely complete, or did not meet our inclusion criteria of combinatorial completeness across multiple environments. We do not claim that our dataset collection is exhaustive, but rather that it provides a diverse and representative foundation for demonstrating our analytical framework. Table 1 summarizes the 12 fitness seascapes used in this study and includes information on the study from which the seascape dataset came, the description of what was measured as a fitness proxy, the number and type of environmental and/or phenotypic contexts in the seascape dataset, and the number of loci in each seascape. Several seascape datasets include log-transformed or normalized versions of phenotypic traits to facilitate interpretation, since most metrics in our framework assume that measurements of fitness are on an additive scale (i.e. that fitness effects combine additively rather than multiplicatively; log-transformation of multiplicative fitness values achieves this; see ***Notation and fitness definition*** below for a detailed discussion). For the purposes of this paper, we provide a detailed discussion of two datasets: the Long-Term Evolution Experiment (LTEE) seascape, which consists of 32 genotypes of *Escherichia coli* generated from the first five beneficial mutations fixed in the LTEE grown in three different conditions (Khan et al. 2011; Flynn et al. 2013), and the *Plasmodium falciparum* DHFR pyrimethamine seascape, which consists of 16 genotypes of *P. falciparum* generated from four important drug-resistant mutations grown in a concentration gradient of the antimalarial drug pyrimethamine (Brown et al. 2010; Ogbunugafor 2022). This choice is intentional, as they represent fitness seascapes from a diversity of organisms, though both are microbial. However, more importantly, they demonstrate two different ways in which seascapes can be constructed: The DHFR pyrimethamine seascape is continuous and quantitative (i.e. the environmental variable (drug concentration) varies along a continuous gradient, though in practice we analyze the seascape at discrete concentration values), while the LTEE seascape is categorical (i.e. the environmental conditions (different growth media) represent qualitatively distinct environments rather than points along a gradient). This demonstrates flexibility in the use cases of the methods we have provided and the greater fitness seascape idea. In addition, we also provide a discussion of two other datasets (Mira et al. 2015; Kosterlitz et al. 2023) in the supplementary information.

**Table 1.** Table of fitness seascapes used in this study.

Dataset (Species)	Fitness proxy	Number of environments/phenotypes	Loci
Human immunodeficiency virus replication (HIV-1) (da Silva et al. 2010)	$\log_{10}\;(\text{replicative capacity})$ of HIV in host cells	**2** (CCR5+ cells, CXCR4+ cells)	5
DHFR proteostasis (abund.) (*Escherichia coli, Listeria grayi, Chlamydia muridarum*) (Guerrero et al. 2019)	ln(Protein abundance) of DHFR variants across different bacterial species and proteostatic backgrounds	**9** (**3** bacterial species (*Escherichia coli*, *Listeria grayi*, *Chlamydia muridarum*) × **3** proteostatic backgrounds (WT, $groEL^{+}$, Δlon))	3
DHFR proteostasis (IC50) (*Escherichia coli, Listeria grayi, Chlamydia muridarum*) (Guerrero et al. 2019)	$\ln\;(IC_{50})$ of DHFR variants across different bacterial species and proteostatic backgrounds	**9** (**3** bacterial species (*Escherichia coli*, *Listeria grayi*, *Chlamydia muridarum*) × **3** proteostatic backgrounds (WT, $groEL^{+}$, Δlon))	3
*Saccharomyces cerevisiae* growth rates (*S. cerevisiae*) (Hall DW et al. 2010)	ln(Growth rate) of *S. cerevisiae*	2 (haploid, diploid)	6
**LTEE** (*E. coli*) (Khan et al. 2011; Flynn et al. 2013)	ln(Relative fitness) of *E. coli*	**3** (DM25, DM25 + EGTA, a calcium chelator, DM25 + guanazole, a ribonucleotide reductase inhibitor)	5
*** ${\text{bla}}_{TEM}$ cefotaxime resistance*** (*E. coli, S. enterica, K. pneumoniae*) (Kosterlitz et al. 2023)	Relative resistance level of ${\text{bla}}_{TEM}$ alleles to cefotaxime in different bacterial species	**3** (*E. coli*, *Salmonella enterica*, *Klebsiella pneumoniae*)	5
DHFR pyrimethamine resistance in *Plasmodium spp.* (*P. falciparum, P. vivax*) (Lozovsky et al. 2009; Jiang et al. 2013)	$\ln\;({\text{Pyrimethamine IC}}_{50})$ in *Plasmodium spp.*	**3** (*P. falciparum* assayed in *E. coli*, *P. vivax* assayed in *E. coli*, *P. vivax* assayed in *S. cerevisiae*)	4
****β*-lactam resistance*** (*E. coli*) (Mira et al. 2015)	ln(Growth rate) of *E. coli* in different β-lactams	**7** (amoxicillin, amoxicillin/clavulanic acid, cefotaxime, cefotetan, cefprozil, ceftazidime, piperacillin/tazobactam)	4
**DHFR resistance in pyrimethamine** (*P. falciparum*) (Brown et al. 2010; Ogbunugafor 2022)	ln(Growth rate) of *P. falciparum* in different concentrations of pyrimethamine	**10** (10 different concentrations of pyrimethamine)	4
DHFR resistance in cycloguanil (*P. falciparum*) (Brown et al. 2010; Ogbunugafor 2022)	ln(Growth rate) of *P. falciparum* in different concentrations of cycloguanil	**10** (10 different concentrations of cycloguanil)	4
Sesquiterpene synthase biochemistry (*Nicotiana tabacum*) (O’Maille et al. 2008)	% production of different products by sesquiterpene synthase mutants in *Nicotiana tabacum*	**4** (premnaspirodiene, 4-epi-eremophilene, 5-epi-aristolochene, minor products)	6
${\text{bla}}_{TEM}$ antibiotic resistance (*E. coli*) (Weinreich et al. 2006; Tan et al. 2011)	$\log_{\sqrt{2}}\;(\text{mean MIC})$ of ${\text{bla}}_{TEM}$ alleles in *E. coli*	3 (cefotaxime, piperacillin, piperacillin + clavulanic acid)	5

Datasets that are discussed in the main section of the paper are bolded, datasets that are discussed in the supplementary information are bolded and italicized.

### Notation and fitness definition

Each dataset consists of a set of genotypes defined over *L* biallelic loci, yielding $2^{L}$ genotypes. A genotype is represented as a binary string $G=(a_{1},a_{2},\ldots ,a_{L})$ where $a_{l}\in \{0,1\}$ denotes the allelic state at locus *l*. Each genotype is measured across *E* environments.

The statistics in our framework, including epistatic coefficients, selection coefficients, and the within-path competition metric $C_{w}$, are defined on an additive scale, where epistasis is a deviation from additivity and selection coefficients are differences. The natural scale for this is Malthusian fitness: the exponential growth rate in continuous-time settings, or ln(W) (where *W* is Darwinian fitness) in discrete-time settings. On this scale, selection coefficients are defined as $s(G^{\prime },G,e)=r(G^{\prime },e)-r(G,e)$, and quantities like $C_{w}$ can be interpreted in terms of sweep times and adaptation speed.

Not all datasets in our study measure Malthusian fitness directly. Some report quantities that are genuine fitness measures or close approximations: the LTEE dataset reports relative Darwinian fitness *w*, from which we use ln(w); the DHFR pyrimethamine and *β*-lactam resistance datasets report log-transformed growth rates, which correspond to Malthusian fitness in a continuous-time growth assay. Others, however, report phenotypic proxies whose relationship to population-level fitness is less direct: $\ln\;(IC_{50})$ in the DHFR proteostasis dataset is a pharmacological property of a protein variant, not a population growth rate; percent product composition in the sesquiterpene synthase dataset is a biochemical phenotype; and $\log_{10}$ or $\log_{\sqrt{2}}$ transformations in other datasets use different bases than the natural logarithm. Table 1 specifies the measured quantity for each dataset.

For datasets where the measured quantity is a genuine fitness measure, the statistics in our framework have their full population-genetic interpretation: epistatic coefficients describe deviations from additive fitness effects, and $C_{w}$ reflects cumulative sweep times along adaptive trajectories. For datasets where a phenotypic proxy is used, the same statistics still provide a valid quantitative description of how the genotype-phenotype map changes across environments; epistatic coefficients describe deviations from additivity on the measured scale, and $C_{w}$ describes how the spacing of phenotypic values along trajectories changes with environment; but the connection to population-dynamic quantities like fixation probabilities or adaptation speed is indirect and depends on the (unmeasured) relationship between the proxy and true fitness. We proceed with this caveat in mind, and restrict our comparative analyses to within-seascape variation across environments, where each dataset is internally consistent regardless of the scale of measurement.

We denote the measured quantity for genotype *G* in environment *e* as r(G,e) throughout, whether it represents Malthusian fitness or a phenotypic proxy, and the difference between genotypes as $s(G^{\prime },G,e)=r(G^{\prime },e)-r(G,e)$. In the equations below, we occasionally use simplified notation (e.g. $r_{i}$ for the value of genotype *i* along a trajectory) for consistency with previously published formulations of each statistic.

### Framework for deconstructing fitness seascapes

**Fig. 1.**
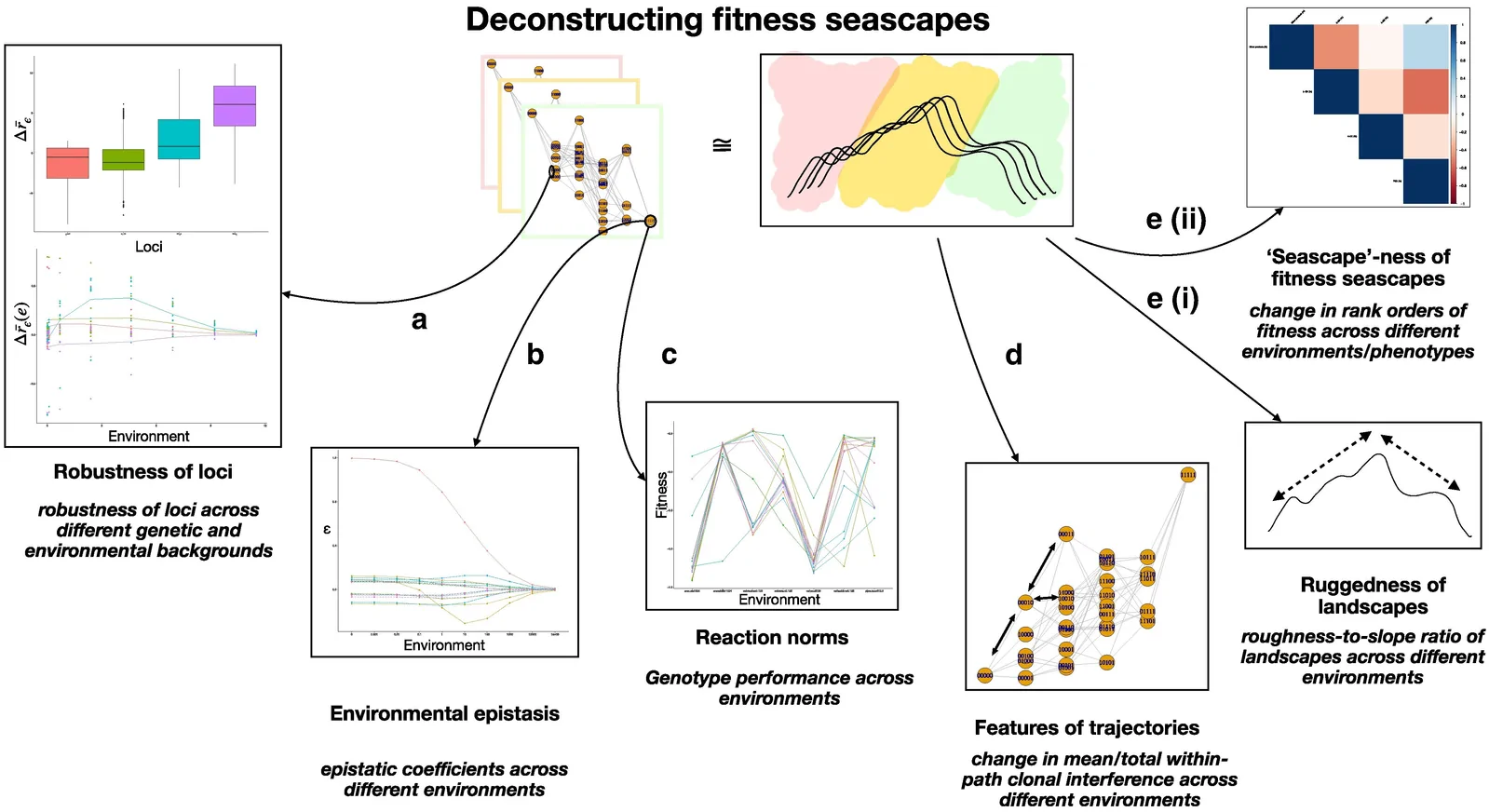
A framework for deconstructing the study of fitness seascapes across different scales of granularity (biological organization). The framework progresses through (from left to right): a) genetic and environmental robustness of loci, b) environmental epistasis at the scale of interactions between loci, c) reaction norms at the scale of alleles, d) the shape of accessible trajectories and within-path competition across those trajectories, and e) (i) ruggedness and (ii) “seascape”-ness to measure topography at the seascape scale.

Figure 1 provides a graphical overview of our proposed framework for studying features of fitness seascapes and how they vary across different contexts; the framework is also summarized in Table 2. Here, we discuss how to compute metrics of different features of fitness seascapes, progressing from loci, to locus interactions, alleles, trajectories, and seascape-wide features.

**Table 2.** A blueprint for studying features of fitness seascapes, discussing in detail the framework illustrated in Fig. 1.

Scale	Description of metric	References
**(a)** Locus	***Robustness ($\Delta {\bar{r}}_{\epsilon }$, $\Delta {\bar{r}}_{\epsilon }(\text{e})$)*** of loci across genetic and/or environmental backgrounds	(Ogbunugafor and Hartl 2016)
**(b)** Locus interaction	Consideration of environmental epistasis, measured by ***mutation-effect reaction norm (MuRN)*** to understand how interactions between loci change across environmental contexts	(Lindsey et al. 2013; Li and Zhang 2018; Hall AE et al. 2019; Ogbunugafor 2022)
**(c)** Allele	***Reaction norm*** to understand how the phenotypic trait values/fitness of individual genotypes vary across different contexts	(Gomulkiewicz and Kirkpatrick 1992; Oomen et al. 2020; Ogbunugafor 2022)
**(d)** Trajectory	***Within-path competition ($C_{w}$)*** to quantify how adaptive trajectories and the speed of adaptive evolution can vary across different environmental contexts	(Ogbunugafor and Eppstein 2016)
**(e)** Landscape topography	∙ **(i) *“Seascape”-ness*** by comparing rank orders of genotypes between any two different environments	(Aita et al. 2001; Szendro et al. 2013; Maltas et al. 2021)
	∙ **(ii) *Roughness-to-slope ratio*** to measure the ruggedness of landscapes and how they vary across environments	
Other theoretical and empirical measures	∙ ***Global epistasis × environment interactions*** to predict the fitness effect of mutations based on genomic and environmental context	(Cvijović et al. 2015; Desponds et al. 2016; Diaz-Colunga et al. 2023; Abreu et al. 2024; Chen P et al. 2024; Petak et al. 2025)
	∙ ***Edge-flip fraction*** to quantify topographical shifts between any two different environments	
	∙ ***Fixation probability of novel mutations*** in fluctuating environments	
	∙ Evolution of ***mutation rates/mutator phenotypes*** in fluctuating environments	
	∙ ***Fitness variance and “environmental memory***” of mutant strains when evolving in fluctuating environments	
	∙ ***Distribution of fitness effects, distribution of diversity (clonal size distributions)*** across different environments	

Here, we discuss the scales of deconstruction, specific metrics measured at every scale, and literature that formally introduced these metrics and related concepts.

We note that the hierarchy that we have imposed is neither essential nor exhaustive. Empirical fitness landscapes/seascapes are not objectively organized into scales based on granularity. Our presentation is largely for organizational purposes. Relatedly, while we have chosen scales and methods for which there are published methods, our organization does not represent every possible sort of analysis applied to a fitness seascape. In fact, it is fair to say that a full examination of every sort of measure of a fitness landscape is far beyond the scope of a single research article; perhaps a future review article can take on this challenge. We urge those interested to apply other analysis methods to understand how environments shape fitness seascape features.

#### Loci: genetic and environmental robustness

At the locus scale, we examine how the average fitness effect of mutations at individual loci varies across genetic backgrounds and environments. We use “robustness” in a specific, operational sense: a locus is *genetically robust* if mutations at that locus have, on average, small fitness effects across all genetic backgrounds and environments, i.e. fitness is relatively insensitive to the allelic state at that locus. A locus exhibits *environmental robustness* if the mean fitness effect of mutations at that locus is consistent across environmental contexts. These definitions are specific to the mutational neighborhood of individual loci within combinatorially complete landscapes, and should be distinguished from other uses of “robustness” in the literature (e.g. mutational robustness of entire genomes, or developmental robustness to environmental perturbation). Adapting from a prior study of reverse evolution in *Plasmodium vivax* DHFR (Ogbunugafor and Hartl 2016), we calculate genetic and environmental robustness as such:

We first calculate the fitness effect of a mutation, ϵ, by taking the mean difference between the fitness of all pairs of allele *j*  ***not*** carrying ϵ and the one-step neighbor carrying mutation ϵ, in a given environment *e*,


(1) $$r_{\;j\epsilon }\left(e\right)-r_{\;j0}\left(e\right),$$

where ϵ corresponds to the mutant allele in a given locus, and $r_{\;j\epsilon }(e)$ and $r_{\;j0}(e)$ denote the Malthusian fitness of the *j*th genetic background with and without mutation ϵ, respectively, in environment *e*. For example, in a 4-locus system, ϵ could refer to one of the following: “1***”, ‘*1**”, “**1*” or “***1”. To calculate the mean robustness of a locus ϵ for a given environment *e*, we compute $\Delta {\bar{r}}_{\epsilon }(e)$:


(2) $$\Delta {\bar{r}}_{\epsilon }\left(e\right)=\frac{1}{2^{L-1}}\sum_{j=1}^{2^{L-1}}r_{\;j\epsilon }\left(e\right)-r_{\;j0}\left(e\right),$$

where *L* denotes the number of loci in the seascape. Genetic robustness is then defined as $\Delta {\bar{r}}_{\epsilon }$ summarized for a locus across all genetic backgrounds ***and*** environmental contexts, whereas environmental robustness is determined by comparing how $\Delta {\bar{r}}_{\epsilon }(e)$ computed for a locus across all genetic backgrounds varies between environmental contexts.

#### Interactions between loci: environmental epistasis and the mutation-effect reaction norm (MuRNs)

Building on individual loci, we can then explore how the interactions between loci (i.e. mutation effects and epistasis (G × G interactions)) shift across contexts. The concept of “environmental epistasis” or environmentally-dependent epistatic interactions have been observed across a number of empirical systems, where the effect of a single mutation, or the magnitude and sign of epistatic interactions between multiple mutations can vary depending on the context in which the system exists (i.e. G × E and G × G × E interactions) (Lindsey et al. 2013; Mira et al. 2015; Hall AE et al. 2019). A tractable way of capturing these interactions (and therefore how variable seascapes are across environments) is through a “mutation-effect reaction norm” (MuRN) (Ogbunugafor 2022). Computing the MuRN for a seascape involves the Walsh-Hadamard transform to compute coefficients corresponding to the sign and magnitude of interactions between mutations (Weinreich et al. 2013, 2018). We refer readers to various studies on the methods and how they have been used to measure higher-order epistasis (Weinreich et al. 2013; Poelwijk et al. 2016) for a more detailed discussion, but we provide a brief summary of the methodology used for computing the MuRN below:

For every environmental context in a given seascape, we compute the single-mutation effect and epistatic interaction coefficients as follows:


(3) $$\gamma =V_{L}H_{L}p,$$

where γ= the Walsh coefficient vector, L= number of loci in the fitness seascape, $V_{L}=$ a normalizing diagonal matrix for $2^{L}$ genotypes, $H_{L}=$ Hadamard matrix for $2^{L}$ genotypes, and p= the vector of fitness values arranged numerically based on genotypes depicted in binary notation. $V_{L}$ and $H_{L}$ are defined recursively as such:


(4) $$V_{L}=\left(\begin{array}{cc} \frac{1}{2}V_{L-1} & 0\\ 0 & -V_{L-1} \end{array}\right),\;V_{0}=1$$

and


(5) $$H_{L}=\left(\begin{array}{cc} H_{L-1} & H_{L-1}\\ H_{L-1} & -H_{L-1} \end{array}\right),\;H_{0}=1.$$

For example, in a 3-locus landscape, the phenotypes in *p* will be arranged in the following manner (corresponding to the respective genotypes): [000,001,010,011,100,101,110,111]. In the MuRN, we can consider not only the coefficients of the individual interactions, but also the mean interaction coefficients within an order to allow for comparison of interactions between orders. This is done by computing the absolute mean coefficient for every order of interaction,


(6) $$\bar{\gamma_{n}}=\frac{\sum_{i=1}^{j}|\gamma_{i}|}{j},$$

where n= the order of interaction, and j= the number of epistatic coefficients that represent the *n*th order of interaction. Each coefficient $\gamma_{i}$ quantifies the contribution of a specific interaction to the fitness of genotypes: first-order coefficients capture the average effect of individual mutations, second-order coefficients capture pairwise epistatic interactions (i.e. the extent to which the combined effect of two mutations deviates from the sum of their individual effects), and higher-order coefficients capture progressively more complex multi-locus interactions. The epistatic “order” thus refers to the number of loci involved in a given interaction. We note that these methods were pioneered for use on fitness landscapes by Daniel Weinreich and colleagues (Weinreich et al. 2013), and similar methods have been utilized in other studies of genotype-phenotype maps (Poelwijk et al. 2016; Doro and Herman 2022; Faure et al. 2024).

#### Alleles: reaction norms

At the allele scale, we can compute reaction norms to describe how the performance (phenotype or fitness) of genotypes is shaped by the environment (Oomen et al. 2020). A reaction norm plots the phenotypic value (here, the fitness proxy such as growth rate, $IC_{50}$, or relative fitness) of a given genotype as a function of the environmental condition. By plotting reaction norms for all genotypes simultaneously, one can visually assess the extent of genotype-by-environment (G×E) interactions: parallel reaction norms indicate consistent genotype rankings across environments, whereas crossing reaction norms indicate that the relative performance of genotypes changes with environment. In the scope of this study, we do a qualitative comparison of how each genotype performs with respect to other genotypes in different environments; however, we note that more quantitative treatments of reaction norms can be and have been utilized in quantitative genetics and phenotypic plasticity, among other fields (Gomulkiewicz and Kirkpatrick 1992; Schlichting and Smith 2002; Lindsey et al. 2013; Oomen and Hutchings 2015). Furthermore, we also take a more quantitative analysis of how the reaction norms manifest at the landscape scales, where we compare how the rank orders of genotypes change across environments (see discussion on landscape topography).

#### Features of trajectories: within-path competition and the speed of evolution

Having examined properties of loci, locus interactions, and alleles, we now look at features at a higher scale of granularity: the evolutionary trajectory. Trajectories represent ordered sequences of mutational steps connecting one genotype to another through the genotype space of a fitness landscape. While they do not necessarily reflect the realized evolutionary path of any particular population, they summarize the set of possible routes that adaptive evolution could follow under strong selection and weak mutation. Nonetheless, molecular evolution does occur along some evolutionary trajectories (or “paths” or “adaptive walks”) more readily than others, and so they are a meaningful scale to examine.

We can consider how adaptive evolutionary trajectories can vary across different contexts as a consequence of topographical changes. Although within-path competition is computed within a single environment rather than under fluctuating conditions, comparing its value across environments reveals how the kinetics of adaptation, and therefore the potential for populations to reach adaptive peaks before the environment shifts, depend on environmental context. In a fluctuating environment, the speed at which a population traverses a landscape in one condition determines how far it progresses before the landscape changes, which is directly relevant to seascape theory (Mustonen and Lässig 2009). We adapt the within-path competition metric, $C_{w}$, proposed in a previous study as a proxy for the speed of adaptive evolution of antimicrobial resistance across landscapes (Ogbunugafor and Eppstein 2016). The formula for computing $C_{w}$ is as follows:


(7) $$C_{w}=\sum_{i=1}^{m-1}\frac{1}{r_{i+1}-r_{i}},$$

where $r_{i}=$ growth rate (or any other fitness proxy) of genotype *i* and m= number of genotypes in a given pathway. We use *r* here rather than *w* (used elsewhere for fitness) to denote that this metric was originally formulated in terms of growth rate; however, *r* can represent any fitness proxy. The subscript *w* in $C_{w}$ stands for “within-path,” denoting that competition is measured among genotypes within a single evolutionary trajectory. We only compute $C_{w}$ for all possible upward trajectories.

To compare how within-path competition varies across different contexts, we generalize $C_{w}$ across the entire landscape, accounting for every possible beneficial trajectory in the landscape, by calculating $C_{w(\text{tot})}$ and $C_{w(\text{avg})}$. Because $C_{w}$ sums inverse selection coefficients along a trajectory, larger values indicate greater cumulative within-path interference and, by extension, slower expected adaptive traversal of that path. Thus $C_{w}$ is inversely related to adaptation speed, where


(8) $$C_{w(\text{tot})}=\sum_{i\in B}\frac{1}{r_{i+1}-r_{i}}$$

for all beneficial mutational steps B across the landscape, and


(9) $$C_{w(\text{avg})}=\frac{1}{\left|B\right|}\sum_{i\in B}\frac{1}{r_{i+1}-r_{i}},$$

where |B|= number of beneficial mutational steps across the landscapes.

#### Topographical features

Finally, at the landscape scale, we can consider how topographical features of seascapes vary across contexts.

##### Topography: ruggedness of fitness seascapes

Given the support for the role of environmental context in shaping seascape features at finer scales (e.g. loci, alleles), we now ask how seascape topography changes in different contexts. One way of measuring the topography of fitness landscapes is to quantify how rugged a landscape is. We can extend this to fitness seascapes by measuring how the ruggedness of landscapes changes across different contexts, using the roughness-to-slope ratio for measuring ruggedness (Aita et al. 2001; Szendro et al. 2013). This is done by fitting a multi-dimensional linear model of fitness to loci:


(10) $$F(x)=\beta_{0}+\sum_{i=1}^{L}\beta_{i}x_{i},$$

where $F(x)=\text{fitness, }x_{i}=\text{state at locus }i,L=\text{number of loci}$. The roughness is the mean squared error of the regression model,


(11) $$\sqrt{\frac{\sum_{x}(f_{x}-F(x){)}^{2}}{m}}\;(\text{where}\;m\;=\text{number of genotypes}),$$

while the slope is the mean of absolute values of linear coefficients,


(12) $$\frac{\sum_{i=1}^{L}|\beta_{i}|}{L}.$$

##### Topography: “seascape-ness” of fitness seascapes

Another way in which we can quantify how much fitness landscape topography fluctuates with respect to the environment (i.e. how ’seascape’-y a dataset is) is to do a pairwise comparison of the rank orders of genotypes in two different contexts, using Kendall’s *τ* rank correlation coefficient. Kendall’s *τ* ranges from −1 to 1, where 1 indicates a perfect agreement of rank orders and −1 a perfect disagreement of rank orders. By computing this matrix of pairwise comparisons, we can see whether the genotype rank orders between pairs of environments highly correlate or do not correlate for a given seascape, and hence whether they can be described more like a static landscape (if all pairs of environments highly correlate) or more like a seascape (if there is variability in how pairs of environments correlate/do not correlate).

#### Relationships among statistics

A key feature of our framework is that the statistics at different scales are not independent, but are connected by the underlying fitness values r(G,e) from which they are all derived. Changes at finer scales necessarily propagate upward. For example, if a shift in environment alters the mean fitness effect of a mutation at a given locus ($\Delta {\bar{r}}_{\epsilon }$), this changes the selection coefficients entering the Walsh-Hadamard decomposition, which can alter epistatic coefficients ($\gamma_{i}$) and thereby reshape the landscape topography captured by ruggedness and rank-order correlations. Conversely, two environments can have identical rank-order correlations (Kendall’s *τ*) while differing substantially in their epistatic structure, because *τ* discards magnitude information that the Walsh-Hadamard coefficients retain.

The trajectory-level metric $C_{w}$ occupies an intermediate position: it depends on selection coefficients between adjacent genotypes along a path, so it is sensitive to both the magnitude and ordering of fitness values but only along specific routes through genotype space. Reaction norms, finally, offer a visual summary of the raw fitness data r(G,e) from which all other statistics are computed; crossing reaction norms are the visual signature of the G×E interactions that the epistatic coefficients quantify. Understanding these dependencies is important for interpreting our results: an observed change in landscape ruggedness across environments is not an independent finding from an observed change in epistatic coefficients, but rather a coarser-grained reflection of the same underlying shifts in the genotype-fitness map.

#### Comparing environmental sensitivity across scales

To compare how strongly environment shapes seascape features at each scale, we compute $\eta_{\mathrm{env}}^{2}$, the fraction of total variance in each statistic attributable to the environment factor. For a statistic *x* computed across multiple entities (loci, epistatic coefficients, genotypes, or trajectories) in multiple environments,


(13) $$\eta_{\mathrm{env}}^{2}=\frac{SS_{\mathrm{env}}}{SS_{\mathrm{total}}}$$

where $SS_{\mathrm{env}}$ is the between-environment sum of squares and $SS_{\mathrm{total}}$ is the total sum of squares. Because $\eta_{\mathrm{env}}^{2}$ is a proportion, it is more readily compared across statistics that are otherwise on different scales.

We compute $\eta_{\mathrm{env}}^{2}$ for: $r_{\;j\epsilon }\left(e\right)-r_{\;j0}\left(e\right)$ across loci and environments (locus scale), individual $\gamma_{i}$ coefficients across interactions and environments (locus-interaction scale), r(G,e) across genotypes and environments (allele scale), and $C_{w}$ across accessible one-step trajectories and environments (trajectory scale). We omit the landscape scale, as the topography statistics (roughness-to-slope ratio, Kendall’s *τ*) yield one value per environment and do not admit the same two-factor decomposition.

## Results

In this section, we discuss how the various properties discussed in our framework vary within and across seascapes. At each scale, we illustrate this by primarily discussing the DHFR pyrimethamine (Brown et al. 2010; Ogbunugafor 2022) and LTEE (Khan et al. 2011; Flynn et al. 2013) seascapes. We also discuss two other seascapes (Mira et al. 2015; Kosterlitz et al. 2023) in Supplementary Information file S1, where all supplementary figures referred to in the main text can be found. We note that the seascapes examined here differ in the nature of their environmental axes: some vary along a quantitative gradient (e.g. drug concentration in the DHFR pyrimethamine seascape), while others represent qualitatively distinct conditions (e.g. different growth media in the LTEE seascape). Consequently, direct quantitative comparisons of the *magnitude* of environmental effects across seascapes should be made cautiously, as the spacing and nature of environmental conditions differ. Our goal is not to rank seascapes by their sensitivity to environmental change, but rather to demonstrate that each seascape exhibits environment-dependent variation at multiple scales of granularity.

### Properties of loci

**Fig. 2.**
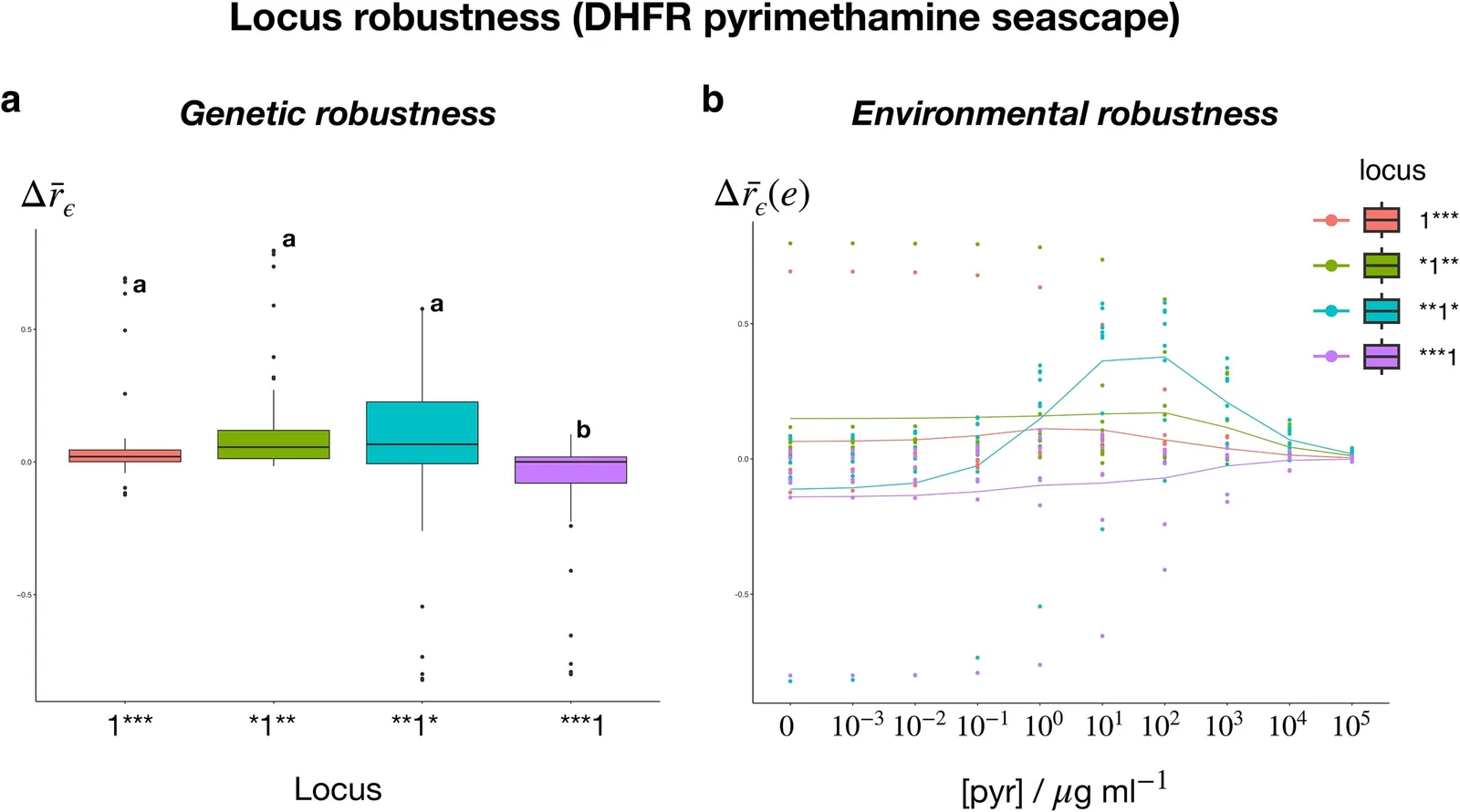
Locus-scale analyses of genetic and environmental robustness for $2^{4}$ DHFR genotypes in *P. falciparum* across a concentration gradient of pyrimethamine (in $\mu {\text{g ml}}^{-1}$) in the DHFR pyrimethamine seascape, where each locus ϵ is represented by a unique color. a) shows a boxplot of $\Delta \bar{r_{\epsilon }}$ for each locus (***genetic robustness***, i.e. $r_{\;j\epsilon }\left(e\right)-r_{\;j0}\left(e\right)$ averaged across all genetic backgrounds and environments for every locus ϵ), with compact letter display of one-way ANOVA ($p=3.61\times 10^{-9}$) and Tukey’s posthoc tests. b) shows $\Delta \bar{r_{\epsilon }}(e)$ for loci averaged across all genetic backgrounds for each of the individual environmental conditions (***environmental robustness***) (i.e. each pyrimethamine concentration; two-way ANOVA: $p=1.37\times 10^{-6}$). Each point represents $r_{\;j\epsilon }\left(e\right)-r_{\;j0}\left(e\right)$ for every combination of ϵ and *e*, and the lines represent mean trend lines for $\Delta \bar{r_{\epsilon }}(e)$ across all environments. See equations 1 and 2 for calculations of $r_{\;j\epsilon }\left(e\right)-r_{\;j0}\left(e\right)$, $\Delta \bar{r_{\epsilon }}(e)$, and $\Delta \bar{r_{\epsilon }}$.

**Fig. 3.**
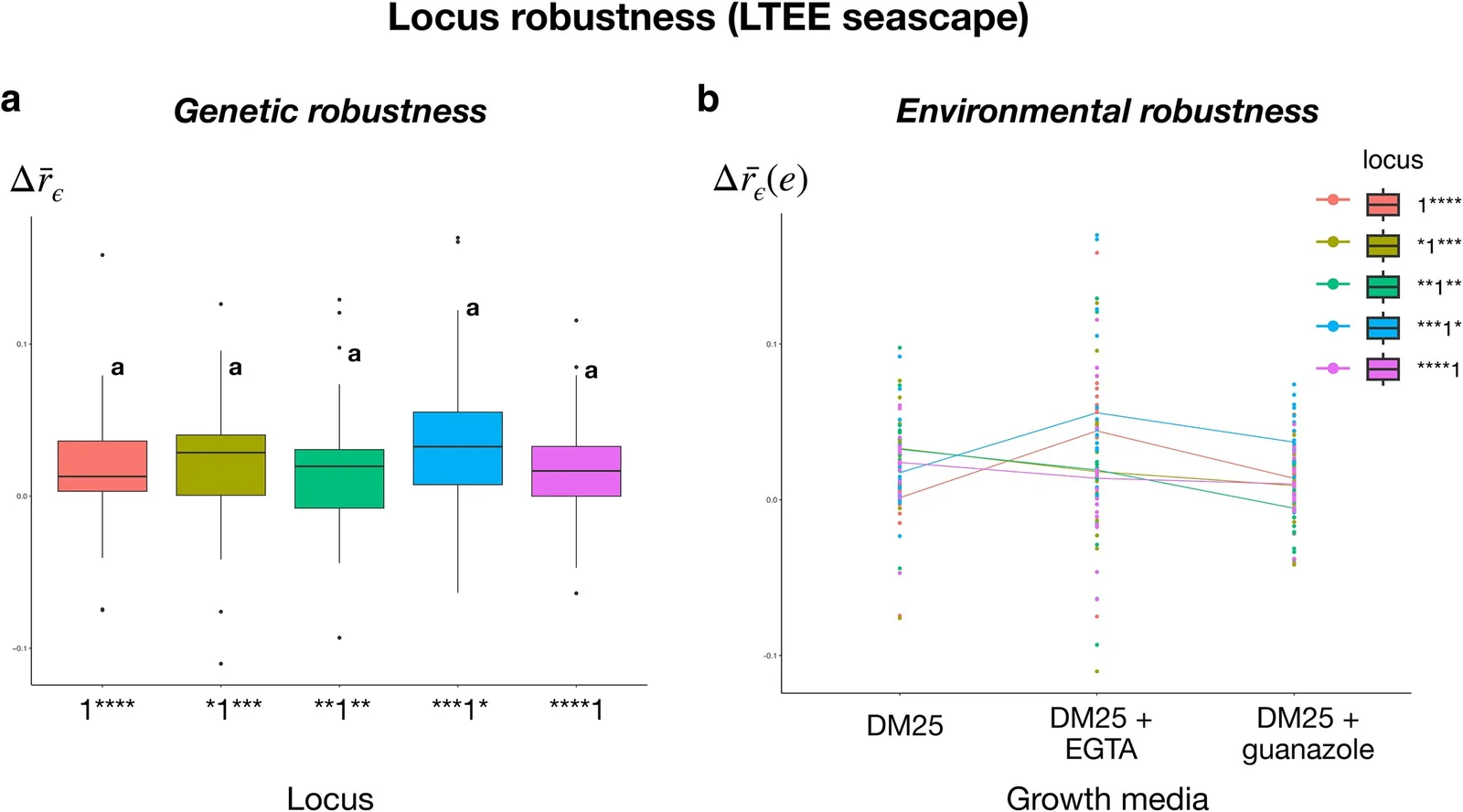
Locus-scale analyses of genetic and environmental robustness for $2^{5}$ genotypes in *E. coli* across 3 growth conditions in the LTEE seascape, where each locus ϵ is represented by a unique color. a) shows a boxplot of $\Delta \bar{r_{\epsilon }}$ for each locus averaged across all genetic backgrounds and environments (***genetic robustness***, i.e. $r_{\;j\epsilon }\left(e\right)-r_{\;j0}\left(e\right)$ averaged across all genetic backgrounds and environments for every locus ϵ), with compact letter display of one-way ANOVA (p=0.0515) and Tukey’s posthoc tests. b) shows $\Delta \bar{r_{\epsilon }}(e)$ for loci averaged across all genetic backgrounds for each of the individual environmental conditions (***environmental robustness***) (i.e. all three growth conditions; two-way ANOVA: $p=2.18\times 10^{-4}$). Each point represents $r_{\;j\epsilon }\left(e\right)-r_{\;j0}\left(e\right)$ for every combination of ϵ and *e*, and the lines represent mean trend lines for $\Delta \bar{r_{\epsilon }}(e)$ across all environments. See Materials and Methods and equations 1 and 2 for calculations of $r_{\;j\epsilon }\left(e\right)-r_{\;j0}\left(e\right)$, $\Delta \bar{r_{\epsilon }}(e)$, and $\Delta \bar{r_{\epsilon }}$.

At the locus scale, we observe that genetic and environmental robustness manifest differently across different seascapes. For example, in the DHFR pyrimethamine seascape, we observe that the fourth locus (I164L) has a lower $\Delta {\bar{r}}_{\epsilon }$ than all the other loci (1-way ANOVA ($p=3.61\times 10^{-9}$) followed by Tukey’s post hoc test, Fig. 2a). However, when considering $\Delta {\bar{r}}_{\epsilon }(e)$ in particular environmental contexts, a two-way ANOVA (fitness∼locus*environment; $p=1.37\times 10^{-6}$) suggests that the third locus (S108N) has a higher $\Delta {\bar{r}}_{\epsilon }(e)$ in pyrimethamine concentrations of $10\;\mu {\text{g ml}}^{-1}(p=0.00521)$, $100\;\mu {\text{g ml}}^{-1}(p=0.00180)$, and $1000\;\mu {\text{g ml}}^{-1}(p=0.0240)$ (Fig. 2b). In the LTEE seascape, we observe that all five loci have similar $\Delta {\bar{r}}_{\epsilon }$ averaged across all genetic backgrounds and environments (1-way ANOVA (p=0.0515) followed by Tukey’s post hoc test, Fig. 3a), but a two-way ANOVA (p=0.0002182) suggests that loci 1 (*rbs*) (p=0.00495) and 4 (*glnUS*) (p=0.00986) have higher $\Delta {\bar{r}}_{\epsilon }(e)$ values than loci 2, 3 and 5 in DM25 + EGTA (Fig. 3). In the *β*-lactam resistance seascape, all four loci have similar $\Delta {\bar{r}}_{\epsilon }$ averaged across all genetic backgrounds and environments (1-way ANOVA, p=0.305), but when we look at $\Delta {\bar{r}}_{\epsilon }(e)$ of the loci for each environment, the mutation G238S in the 3rd locus has a higher $\Delta {\bar{r}}_{\epsilon }(e)$ in certain environments, as demonstrated through a two-way ANOVA that examined the effect of locus and environment on $\Delta {\bar{r}}_{\epsilon }(e)$ ($[\text{cefotaxime}{]}_{0.123\;\mu {\text{g ml}}^{-1}}$  $*\text{G238S}\;:\;p=1.96\times 10^{-5},$  $[\text{cefotetan}{]}_{0.125\;\mu {\text{g ml}}^{-1}}\;*\;\text{G238S}\;:\;p=0.00404,[\text{cefprozil}{]}_{128\;\mu {\text{g ml}}^{-1}}*$  $\text{G238S}\;:\;p=0.01373,[\text{ceftazidime}{]}_{0.125\;\mu {\text{g ml}}^{-1}}*\text{G238S}\;:\;p=0.00353$, Supplementary Fig. S1a and b). On the other hand, in the ${\text{bla}}_{TEM}$ cefotaxime resistance seascape, we observe that of the 5 loci, the mutation G238S in the 5th locus has the highest $\Delta {\bar{r}}_{\epsilon }$, followed by the mutation E104K in the 3rd locus; these differences in $\Delta {\bar{r}}_{\epsilon }$ compared to the other loci are statistically significant (1-way ANOVA followed by Tukey’s post hoc test, Supplementary Fig. S2a) and this holds across the three different species environments (*Escherichia coli*, *Klebsiella pneumoniae*, *Salmonella enterica*) (2-way ANOVA: locus, $p<2.2\times 10^{-16}$; species, p=0.412; locus*species, p=0.966, Supplementary Fig. S2b).

### Properties of interactions between loci

**Fig. 4.**
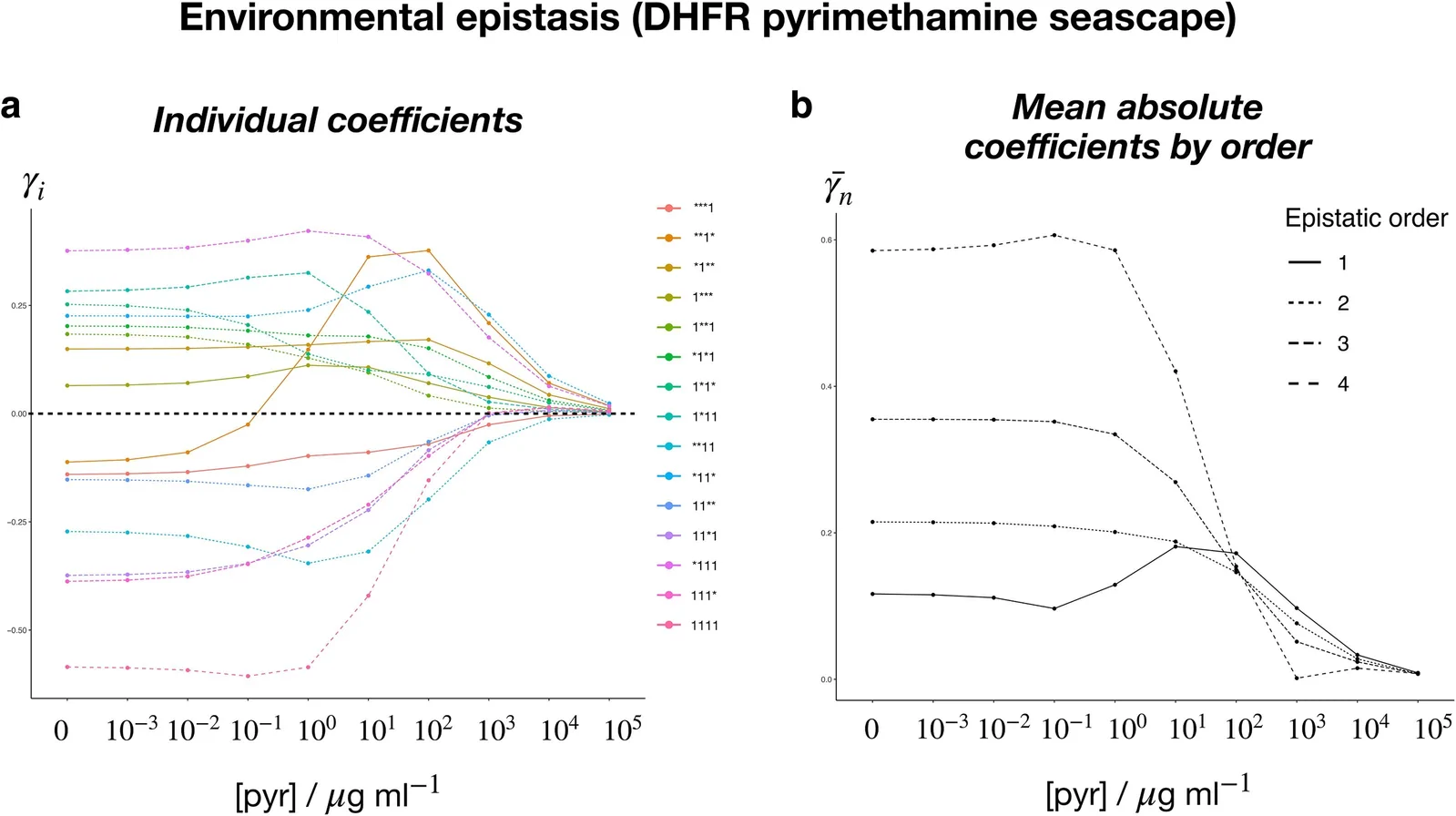
Locus-interaction analyses of environmental epistasis, as computed through the mutation-effect reaction norm (MuRN), for $2^{4}$ DHFR genotypes in *P. falciparum* across a concentration gradient of pyrimethamine (in $\mu {\text{g ml}}^{-1}$) in the DHFR pyrimethamine seascape. Interaction corresponding to each coefficient is defined by the 1s in the binary description (e.g. *11* refers to a 2nd-order interaction between loci 2 and 3, whereas 1*11 refers to a 3rd-order interaction between loci 1, 3 and 4). a) shows the MuRN depicting coefficients of every possible epistatic interaction ($\gamma_{i}$) (with a black horizontal dashed line to indicate $\gamma_{i}=0$). Each interaction is represented by a different colored line, and the order of a given interaction is represented by how dashed a line is (1st order: solid line, increasingly shorter dashes as order increases). b) shows the absolute mean of epistatic coefficients based on epistatic order ($\bar{\gamma_{n}}$), with 1st order represented by a solid line, and higher order interactions being represented by dashed lines (increasingly shorter dashes as order increases). Note that for all figures, the 0th-order interaction is not shown. See equations 3 and 6 for calculations of $\gamma_{i}$ and $\bar{\gamma_{n}}$.

**Fig. 5.**
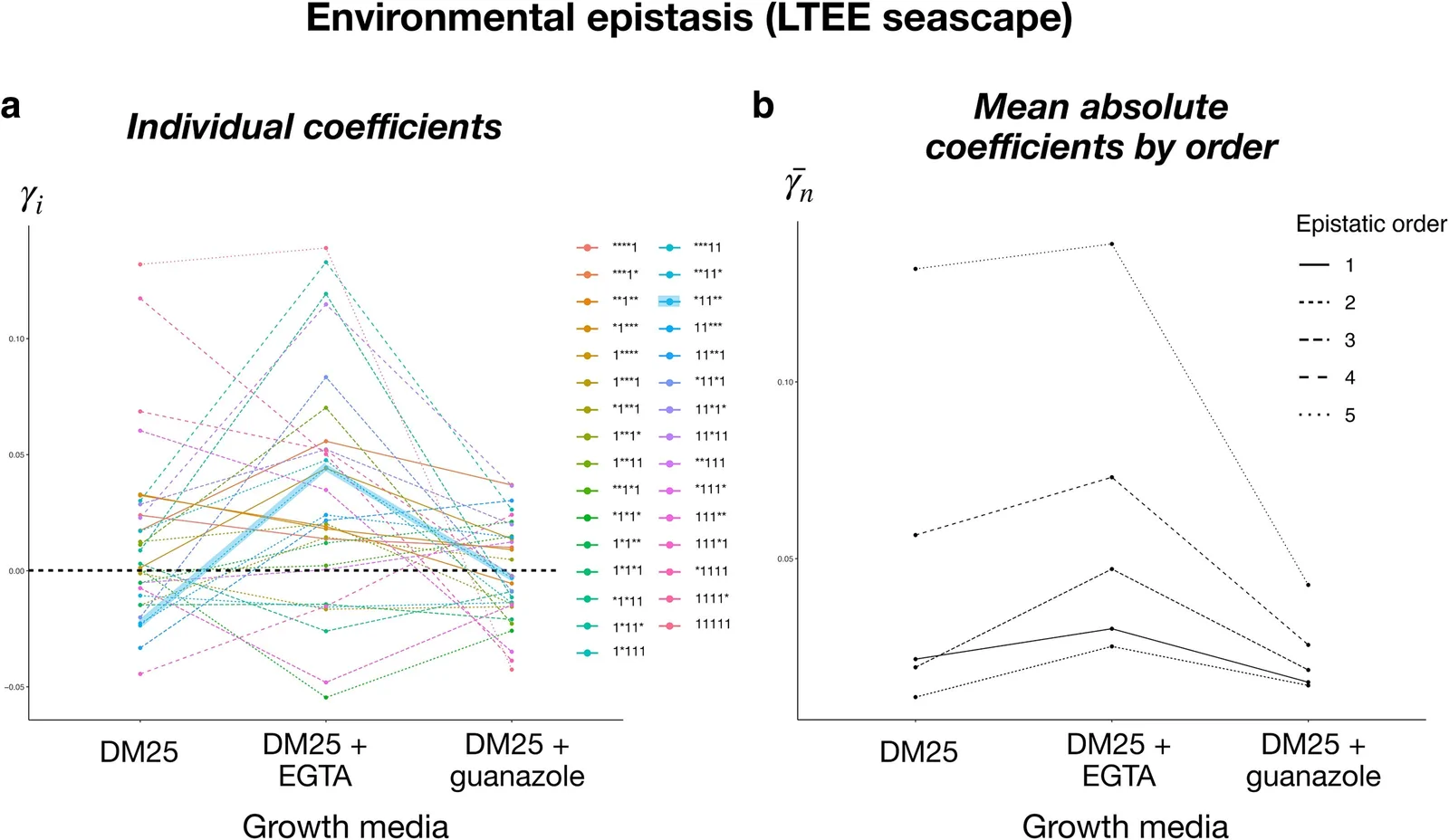
Locus-interaction analyses of environmental epistasis, as computed through the mutation-effect reaction norm (MuRN), for $2^{5}$ genotypes in *E. coli* across 3 growth conditions in the LTEE seascape. Interaction corresponding to each coefficient is defined by the 1s in the binary description (e.g. *11** refers to a 2nd-order interaction between loci 2 and 3, whereas 1*11* refers to a 3rd-order interaction between loci 1, 3 and 4). a) shows the MuRN depicting coefficients of every possible epistatic interaction ($\gamma_{i}$) (with a black horizontal dashed line to indicate $\gamma_{i}=0$ and the *11** pairwise interaction highlighted to demonstrate an example of an epistatic interaction changing in sign and magnitude across different environments). Each interaction is represented by a different colored line, and the order of a given interaction is represented by how dashed a line is (1st order: solid line, increasingly shorter dashes as order increases). b) shows the absolute mean of epistatic coefficients based on epistatic order ($\bar{\gamma_{n}}$), with 1st order represented by a solid line, and higher order interactions being represented by dashed lines (increasingly shorter dashes as order increases). Note that for all figures, the 0th-order interaction is not shown. See equations 3 and 6 for calculations of $\gamma_{i}$ and $\bar{\gamma_{n}}$.

At the scale of locus interactions, the mutation effect reaction norms (MuRNs) demonstrate how mutation effects and epistatic interactions vary across environmental contexts. Across the seascapes, we observe that the MuRNs for individual coefficients ($\gamma_{i}$) change in slope across environments, sometimes resulting in MuRNs intersecting (e.g. $\gamma_{**1*}$ and $\gamma_{*111}$ MuRNs intersecting at $10\text{ and }100\;\mu {\text{g ml}}^{-1}$ in the DHFR pyrimethamine seascape), suggesting the presence of G × G × E interactions in the genotype-phenotype map (Figs. 4a and 5a; Supplementary Figs. S3a and S4a) (Ogbunugafor 2022). Additionally, in the DHFR pyrimethamine seascape, $\bar{\gamma_{n}}$ is observed to be highest for the 4th order interaction, followed by 3rd, 2nd, and 1st-order at lower concentrations of pyrimethamine, but at concentrations of $100\;\mu {\text{g ml}}^{-1}$ and above, $\bar{\gamma_{n}}$ is highest for 1st order interactions, followed by 2nd, 3rd, and then 4th order, with the magnitudes eventually converging (Fig. 4b). This convergence at high pyrimethamine concentrations likely reflects the pharmacodynamic saturation of the drug-enzyme interaction: at sufficiently high drug concentrations, only the most resistant genotypes maintain appreciable fitness, compressing the fitness differences among genotypes and thereby reducing the magnitude of all epistatic coefficients toward similar values. 5th and 4th order interactions also have a higher $\bar{\gamma_{n}}$ across all environments in the LTEE seascape (Fig. 5b). In the *β*-lactam resistance seascape, the magnitude of the coefficient ($\bar{\gamma_{n}}$) for the 4th order interaction is highest in all environments except for amoxicillin ($1,024\;\mu {\text{g ml}}^{-1}$)/clavulanic acid (Supplementary Fig. S3b), thus demonstrating how higher-order interactions can shape fitness landscapes across different environmental contexts. In the ${\text{bla}}_{TEM}$ cefotaxime resistance seascape, the largest $\gamma_{i}$ is attributed to the single mutation G238S in locus 5, thus mirroring the locus-scale analysis. However, the MuRN also uncovers how the sign and magnitude of pairwise and other higher-order interaction effects fare in comparison to the single locus mutation effects across environments; in particular, in *S. enterica*, the magnitude of the coefficient for the 5th order interaction is higher than $\bar{\gamma_{n}}$ for 1st to 4th order interactions, whereas in *E. coli* and *K. pneumoniae*, the 5th order interaction has the lowest magnitude of all $\bar{\gamma_{n}}$ (Supplementary Fig. S4b). All in all, these analyses demonstrate how interactions between loci can differ in sign and magnitude and therefore structure fitness landscapes across different environmental contexts.

### Properties of alleles

**Fig. 6.**
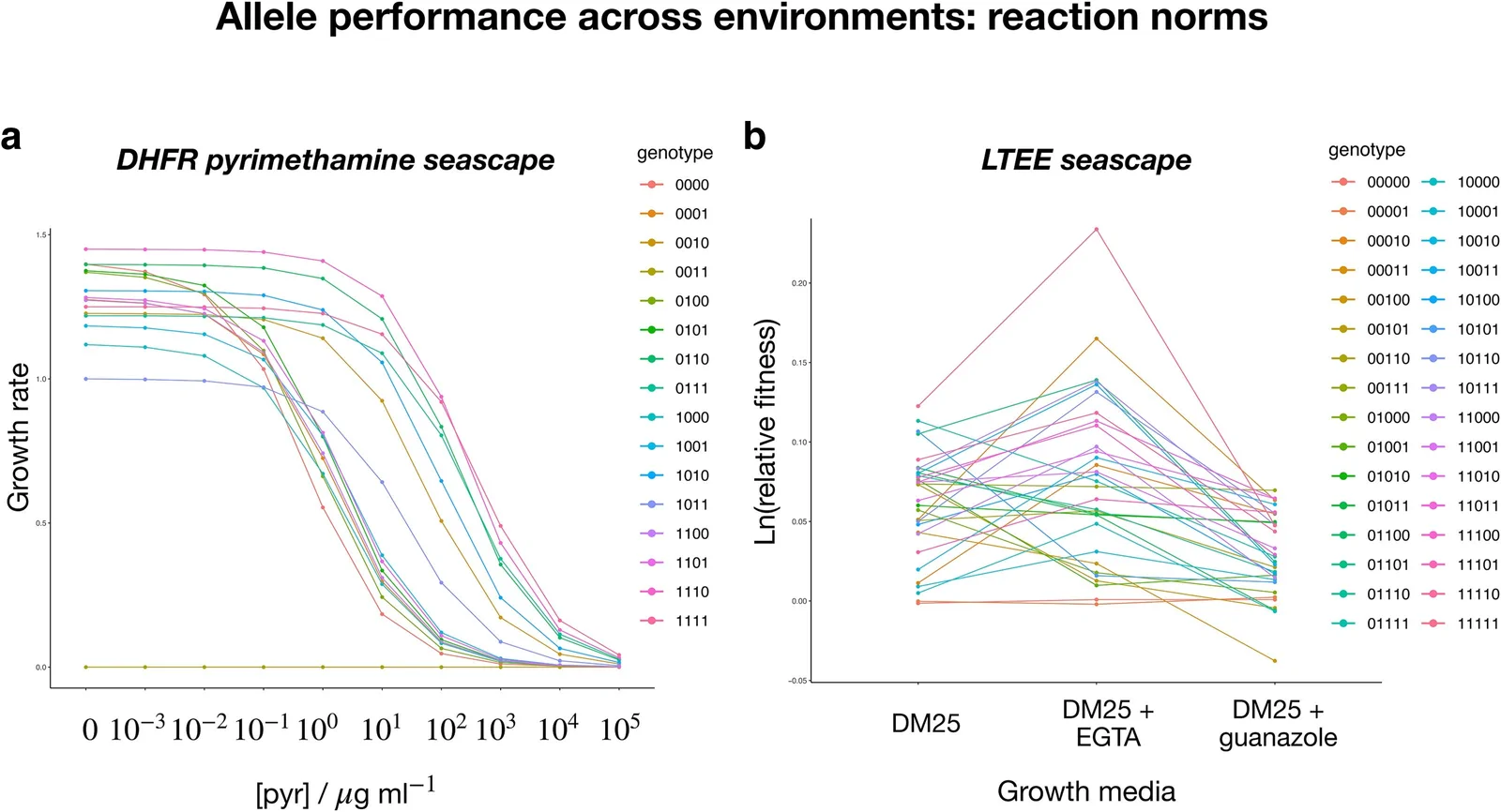
Allele performance across environments: reaction norms. Genotype performance in every environment is represented by a differently colored line for each genotype. a) Reaction norms of DHFR pyrimethamine seascape, demonstrating the growth rate of $2^{4}$ DHFR genotypes in *P. falciparum* across a concentration gradient of pyrimethamine (in $\mu {\text{g ml}}^{-1}$) (Brown et al. 2010; Ogbunugafor 2022), b) Reaction norms of LTEE seascape, demonstrating ln(relative fitness) of $2^{5}$  *E. coli* genotypes from the Long-Term Evolution Experiment in 3 different growth conditions (Khan et al. 2011; Flynn et al. 2013).

Across seascapes, the *relative* performance of alleles can vary across environmental contexts (Fig. 6; Supplementary Fig. S5). Even in seascapes where the rank orders of most alleles remain mostly consistent across environments (e.g. Supplementary Fig. S5b), there are still examples of alleles that change in relative rank order under different environmental contexts, which we discuss in further detail in the next section on landscapes-scale analyses. As well articulated in the literature on reaction norms, the slope and interactions between lines indicate features of the underlying genotype-phenotype map (Sae-Lim et al. 2016). For example, crossing reaction norms are representative of (G×E) interactions, which are widespread in both the DHFR pyrimethamine and LTEE fitness seascapes (Fig. 6), thus highlighting how the structure of genotype-fitness maps is shaped by interactions between the genotype and the environment.

### Properties of trajectories

**Fig. 7.**
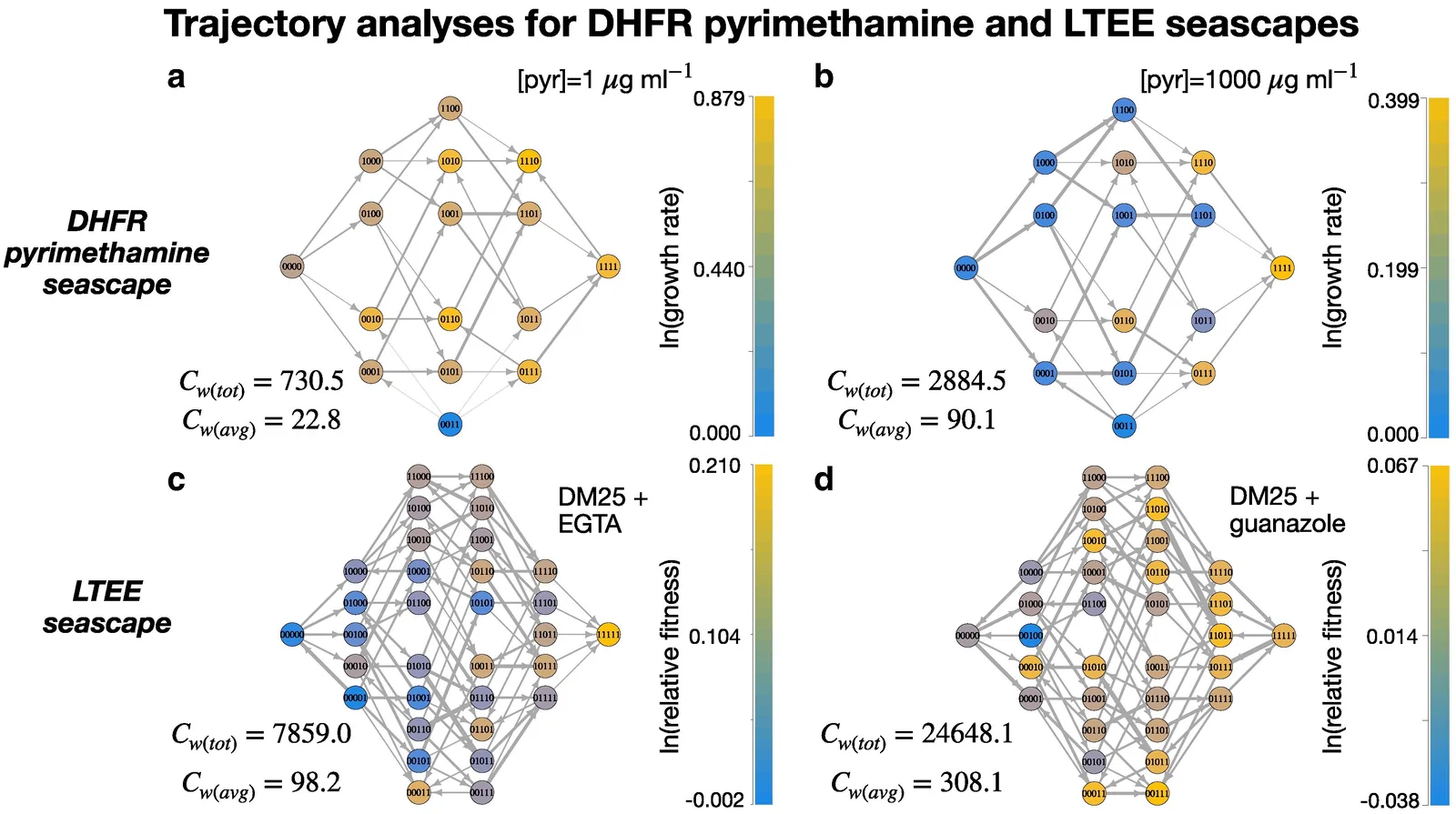
Trajectory-scale analyses of within-path competition for the DHFR pyrimethamine (top row) and LTEE seascapes (bottom row), where the fitness landscapes are depicted as hypercubes. Each vertex depicts a genotype, and each edge connects two genotypes that are one mutational step away from each other. Vertices are arranged from left to right in increasing Hamming distance from the wild-type genotype (i.e. number of mutations from wild-type), and are color-coded according to their fitness values (low fitness: darker shade; high fitness: lighter shade). Edge width is determined by the $C_{w}$ of the step between two given genotypes/nodes, with thinner edges indicating smaller $C_{w}$ and thicker edges indicating larger $C_{w}$ for a given step. Note that vertex color and edge width scales vary for each seascape and environmental context. a) Hypercube with $C_{w(\text{avg})}$ and $C_{w(\text{tot})}$ values for the DHFR pyrimethamine seascape at a pyrimethamine concentration of $1\;\mu {\text{g ml}}^{-1}$. b) Hypercube with $C_{w(\text{avg})}$ and $C_{w(\text{tot})}$ values for the DHFR pyrimethamine seascape at a pyrimethamine concentration of $1,000\;\mu {\text{g ml}}^{-1}$. c) Hypercube with $C_{w(\text{avg})}$ and $C_{w(\text{tot})}$ values for the LTEE seascape in DM25 + EGTA. d) Hypercube with $C_{w(\text{avg})}$ and $C_{w(\text{tot})}$ values for the LTEE seascape in DM25 + guanazole. Panels (a) and (b) illustrate how within-path competition shifts across a drug concentration gradient: at a lower concentration of pyrimethamine, fitness differences between many adjacent genotypes are larger, yielding lower $C_{w}$ (faster expected traversal), whereas at a higher concentration the fitness differences between most genotypes are smaller, resulting in higher $C_{w}$ outside of a select few trajectories. Panels (c) and (d) show that even qualitatively distinct environments (different growth media supplements) can produce different $C_{w}$ distributions across trajectories. See equations 7–9 for calculations of $C_{w(\text{avg})}$ and $C_{w(\text{tot})}$.

Doing a qualitative comparison of trajectories across seascapes, we observe that the distributions of $C_{w(\text{tot})}$ and $C_{w(\text{avg})}$ differ across seascapes, and within each seascape, different environmental contexts can give rise to different $C_{w(\text{tot})}$ and $C_{w(\text{avg})}$ values (Fig. 7; Supplementary Fig. S6a and b). We note that these comparisons are qualitative; formal statistical tests are not straightforward here because the $C_{w}$ values are computed over all accessible trajectories in a landscape, and these trajectories are not independent of one another (they share mutational steps). We therefore limit our interpretation to describing observed differences rather than making claims of statistical significance. This suggests that the average and/or total within-path competition across all possible evolutionary trajectories can vary depending on the environment, which has broader implications for understanding how adaptation rates across the landscape are dependent on the environment.

### Properties of seascape topography

**Fig. 8.**
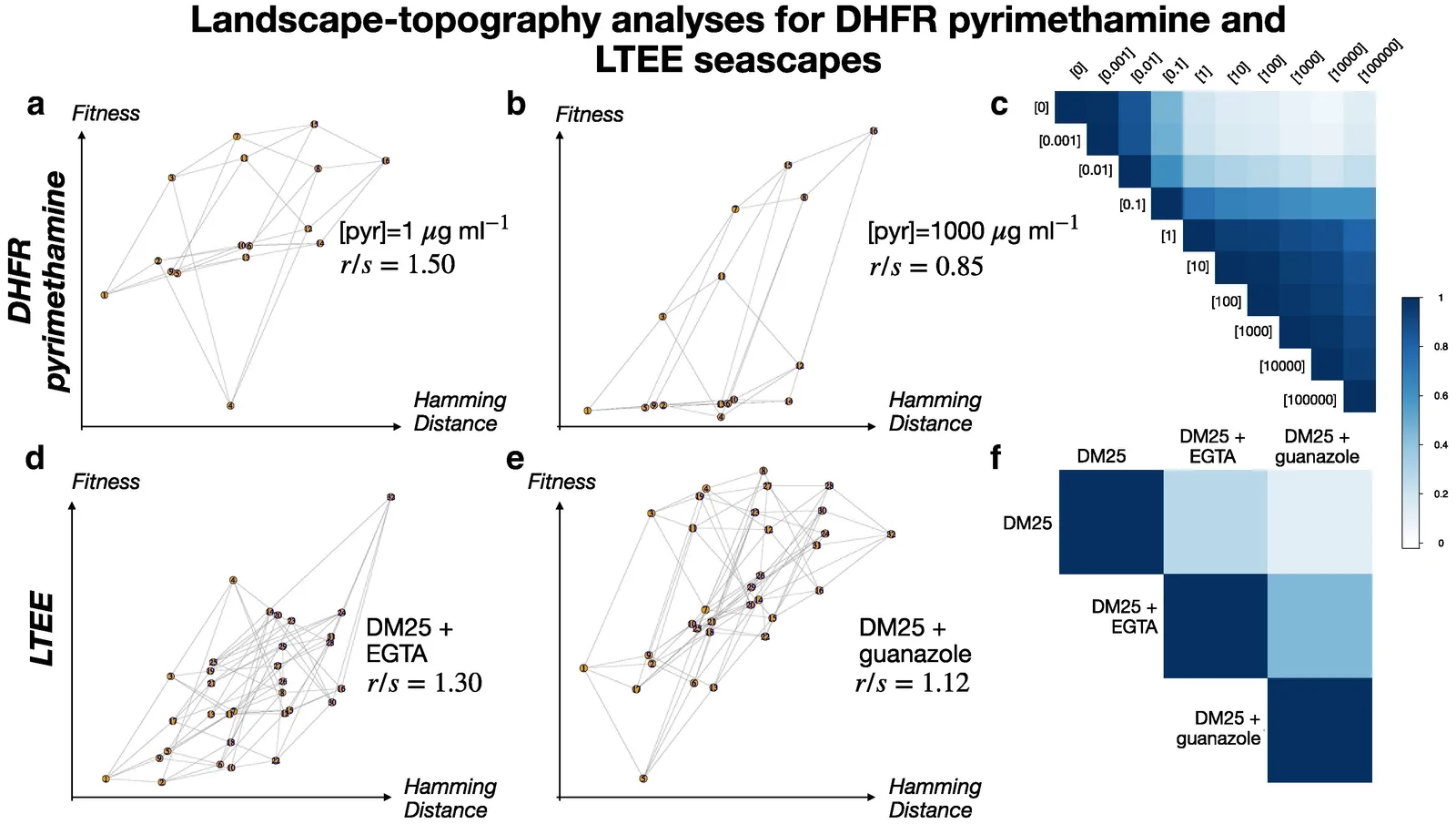
Topography-scale analyses for the DHFR pyrimethamine (top row) and LTEE (bottom row) seascapes. In panels a, b, d, e, each network graph depicts a particular landscape (i.e. seascape dataset for a given environmental context), where each node represents a genotype and the edges connect genotypes separated by one mutational step. The *x*-axis denotes the Hamming distance of genotypes from the wild-type genotype (i.e. number of mutations from wild-type), while the *y*-axis denotes the fitness of the genotype. Note that the scales of the *x*-axis and y-axis for the fitness graphs are not the same across the four panels; they are drawn with respect to the number of loci (*x*-axis) and the relative fitness of the genotypes in the dataset and particular environmental context. Additionally, coordinates of nodes are jittered to ensure that individual genotypes can be visualized distinctly in the network graph. (a,b,d,e) Roughness-to-slope ratios (r/s) for the DHFR pyrimethamine seascape at pyrimethamine concentrations of $1\;\mu {\text{g ml}}^{-1}$ and $1,000\;\mu {\text{g ml}}^{-1}$, and for the LTEE seascape in DM25 + EGTA and DM25 + guanazole, respectively. See Supplementary Table S1 in Supplementary File S1 for how node numbers correspond to genotypes in each seascape. c) Kendall’s *τ* rank-order correlation matrix for the DHFR pyrimethamine seascape across the concentration gradient. Closely spaced concentrations show high positive correlations (darker shades), indicating that the rank ordering of genotypes is largely preserved across nearby concentrations, while more distant concentrations show weaker correlations (lighter shades). f) Kendall’s *τ* correlation matrix for the LTEE seascape across three growth conditions. All three environments show high positive rank-order correlations (darker shades), indicating that the relative performance of genotypes is broadly consistent across these categorical environments.

Finally, we qualitatively compare topographical features across the seascapes. As with the trajectory-scale analyses, we present these comparisons descriptively rather than with formal statistical tests, given the interdependence of the underlying data structures. When comparing topographical features, we observe that the distributions of roughness-to-slope ratios look different across seascapes, and that within each seascape, environmental contexts can give rise to different extents of ruggedness (Fig. 8a, b, d, e (see also Supplementary Table S1 for Fig. 8 node legend); Supplementary Fig. S8). Furthermore, the Kendall’s *τ* correlation matrices (capturing “seascape”-ness) are different across the 12 seascapes. In some seascapes, the rank orders are highly positively correlated across environments, for example, pyrimethamine concentrations close to each other in magnitude in the DHFR seascape, across the three growth media conditions in the LTEE seascape, and across the three species in the ${\text{bla}}_{TEM}$ cefotaxime resistance seascape (Fig. 8c and f; Supplementary Fig. S7b). In other contexts, the rank orders are less positively correlated and/or sometimes even negatively correlated between environments such as in the *β*-lactam resistance seascape (Supplementary Fig. S7a), potentially suggesting trade-off relationships in adapting to different types of *β*-lactams and/or different concentrations of *β*-lactams (Mira et al. 2015; Guerrero et al. 2024).

### Environmental sensitivity across scales of granularity

The preceding analyses demonstrate that environmental context shapes seascape features at every scale we examined. To compare the relative sensitivity of each scale to environmental variation, we compute $\eta_{\mathrm{env}}^{2}$ as described in the Materials and Methods (Eq. 13).

Across the DHFR pyrimethamine seascape, we find that $\eta_{\mathrm{env}}^{2}$ varies substantially across scales (Supplementary Fig. S9, Table 3). At the allele scale, $\eta_{\mathrm{env}}^{2}=0.72$ ($p<10^{-15}$), indicating that roughly three-quarters of the total variance in genotype fitness values is attributable to drug concentration. At the locus scale (robustness), $\eta_{\mathrm{env}}^{2}=0.052$ (p=0.053), indicating that the mean fitness effect of individual mutations varies far more across genetic backgrounds than across environments. The locus-interaction scale shows even lower environmental sensitivity ($\eta_{\mathrm{env}}^{2}<0.01$, p=1.0): the epistatic coefficients are largely determined by the structure of genetic interactions rather than the drug concentration. The trajectory scale, by contrast, is highly environmentally sensitive ($\eta_{\mathrm{env}}^{2}=0.22$, $p<10^{-12}$), reflecting the fact that the number and identity of accessible trajectories, and the $C_{w}$ values along them, shift dramatically across the concentration gradient. In the LTEE seascape, which has only three categorical environments, the allele, locus and interaction scales show a small but statistically significant environmental effect (allele: $\eta_{\mathrm{env}}^{2}=0.21$, $p<10^{-4}$; locus: $\eta_{\mathrm{env}}^{2}=0.032$, p=0.021; interaction: $\eta_{\mathrm{env}}^{2}=0.12$, p=0.0033), but not the trajectory scale ($\eta_{\mathrm{env}}^{2}<0.01,p=0.39$). This suggests that the environment has a noticeable effect on the fitness of loci and alleles, as well as the structure of epistatic interactions, but not as strong an effect on the number and identity of accessible trajectories. Across all the seascapes, the environmental effect at the allele scale is statistically significant in 10 of 12 seascapes. At the locus-interaction scale, the effect is nonsignificant in 11 of 12 seascapes (the exception being the LTEE seascape; $\eta_{\mathrm{env}}^{2}=0.12$, p=0.0033). At the locus and trajectory scales, the effect is statistically significant in 5 of 12 seascapes.

**Table 3.** Environmental sensitivity across scales of granularity for all seascapes.

Seascape	Allele	Locus	Interaction	Trajectory
DHFR pyrimethamine	**0.72** $\left(F_{9,150}=43.15,\;p<10^{-15}\right)$	0.052 $\left(F_{9,310}=1.886,\;p=0.053\right)$	<0.01 $\left(F_{9,150}=0.103,\;p=1\right)$	**0.22** $\left(F_{9,299}=9.54,\;p<10^{-12}\right)$
DHFR cycloguanil	**0.65** $\left(F_{9,150}=31.17,\;p<10^{-15}\right)$	**0.074** $\left(F_{9,310}=2.74,\;p=0.0043\right)$	<0.01 $\left(F_{9,150}=0.120,\;p=1\right)$	**0.25** $\left(F_{9,310}=11.5,\;p<10^{-14}\right)$
LTEE	**0.21** ($F_{2,93}=12.22,\;p<10^{-4}$)	**0.03** ($F_{2,237}=3.917,\;p=0.021$)	**0.12** ($F_{2,93}=6.09,\;p=0.0033$)	<0.01 ($F_{2,237}=0.955,\;p=0.39$)
*β*-lactam resistance	**0.56** ($F_{6,105}=22.24,\;p<10^{-15}$)	0.038 ($F_{6,217}=1.433,\;p=0.20$)	0.024 ($F_{6,105}=0.432,\;p=0.86$)	0.032 ($F_{6,215}=1.177,\;p=0.32$)
${\text{bla}}_{TEM}$ cefotaxime	0.02 ($F_{2,93}=0.866,\;p=0.42$)	<0.01 ($F_{2,237}=0.263,\;p=0.77$)	<0.01 ($F_{2,93}=0.187,\;p=0.83$)	<0.01 ($F_{2,218}=0.898,\;p=0.41$)
HIV replication	**0.70** ($F_{1,62}=145.8,\;p<10^{-15}$)	**0.12** ($F_{1,158}=20.73,\;p<10^{-4}$)	0.012 ($F_{1,62}=0.754,\;p=0.39$)	0.011 ($F_{1,158}=1.818,\;p=0.18$)
*S. cerevisiae*	0.01 ($F_{1,126}=1.91,\;p=0.17$)	<0.01 ($F_{1,382}=0.070,\;p=0.79$)	<0.01 ($F_{1,126}=0.282,\;p=0.60$)	<0.01 ($F_{1,382}=1.074,\;p=0.30$)
DHFR proteo. (${\text{IC}}_{50}$)	**0.57** ($F_{8,63}=10.49,\;p<10^{-8}$)	0.10 ($F_{8,99}=1.435,\;p=0.19$)	0.037 ($F_{8,63}=0.299,\;p=0.96$)	0.039 ($F_{8,99}=0.4965,\;p=0.86$)
DHFR proteo. (abund.)	**0.51** ($F_{8,63}=8.225,\;p<10^{-6}$)	0.077 ($F_{8,99}=1.034,\;p=0.42$)	<0.01 ($F_{8,63}=0.034,\;p=1$)	0.088 ($F_{8,99}=1.194,\;p=0.31$)
Sesquiterpene synthase	**0.40** ($F_{3,252}=56.29,\;p<10^{-15}$)	**0.038** ($F_{3,764}=9.93,\;p<10^{-5}$)	<0.01 ($F_{3,252}=0.782,\;p=0.50$)	**0.026** ($F_{3,763}=6.89,\;p<10^{-3}$)
DHFR pyr *Plasm.* spp.	**0.19** ($F_{2,45}=5.357,\;p=0.008$)	<0.01 ($F_{2,93}=0.1271,\;p=0.88$)	0.018 ($F_{2,45}=0.4204,\;p=0.66$)	**0.063** ($F_{2,93}=3.133,\;p=0.048$)
${\text{bla}}_{TEM}$ antibiotic resistance	**0.44** ($F_{2,93}=37.27,\;p<10^{-11}$)	**0.24** ($F_{2,237}=37.65,\;p<10^{-14}$)	0.022 ($F_{2,93}=1.056,\;p=0.35$)	**0.079** ($F_{2,184}=7.891,\;p<10^{-3}$)

For each seascape and scale, we report $\eta_{\mathrm{env}}^{2}$ (fraction of variance attributable to environment), *F*-statistic, and *p*-value from a one-way ANOVA with environment as the factor. Significant results (p<0.05) are indicated in bold. Note that statistical power differs across scales due to unequal numbers of observations (see text for discussion).

Several caveats apply to this comparison and should caution against over-interpretation. These results describe the 12 seascapes examined in this study and should not be treated as universal generalizations about fitness seascapes. Most fundamentally, the statistics at different scales are derived from the same underlying fitness values through different transformations, and these transformations can amplify or dampen environmental variation to different degrees. For example, $C_{w}$ involves reciprocals of fitness differences, which can magnify small environmental shifts in selection coefficients into large changes in $C_{w}$; conversely, $\Delta {\bar{r}}_{\epsilon }$ averages fitness effects across genetic backgrounds, which can mask environment-specific variation. The $\eta_{\mathrm{env}}^{2}$ values are therefore not on a common footing across scales, and the ranking of scales by $\eta_{\mathrm{env}}^{2}$ should be interpreted as suggestive rather than definitive. Additionally, statistical power differs across scales: the allele and interaction scales have $2^{L}\times E$ observations per seascape, while the locus scale has $2^{L-1}\times L\times E$ observations and the trajectory scale $\approx 2^{L-1}\times L\times E$ observations per seascape. Nevertheless, that the environmental effect on the allele scale is statistically significant across nearly all seascapes but the environmental effect on the locus interaction scale is not statistically significant across nearly all seascapes despite both scales having the same number of observations provides stronger evidence that epistatic structure is relatively insensitive to environment.

The decomposition also treats all environments as exchangeable categories, which is appropriate for the LTEE but ignores the ordered structure of the pyrimethamine concentration gradient. Finally, the trajectory-scale results are influenced by the fact that the number of accessible paths itself changes across environments, which conflates two distinct effects: changes in $C_{w}$ along existing paths and the appearance or disappearance of paths entirely. Despite these limitations, the decomposition provides a first empirical comparison of how environmental variation partitions across different aspects of the genotype-fitness map in these systems.

## Discussion

In this study, we examine a set of empirical fitness seascapes with respect to how features vary across environments of various kinds. In doing so, we address an important question: to what extent does the fitness seascape alter our picture of evolutionary genetics? And does the added environmental context offer meaningful information on the dynamics of evolution at the molecular scale? Or is it mostly superfluous? To address this, we compare features of seascapes at multiple scales of granularity: from loci and mutation effects, to alleles, trajectories, and global features of seascape topography. In order to appreciate the importance of the findings, we organize them in order of magnitude of scale, starting with loci, followed by locus interactions, alleles, trajectories, and landscapes. We find that within individual seascapes and across seascapes, these features change appreciably depending on environmental context, thus highlighting the importance of (a) pivoting from the landscape to the seascape metaphor and (b) studying how these features can vary at different scales of biological organization. In doing so, we advocate for a “blueprint” for studying features of fitness seascapes across different scales of biological organization, which we summarize in Fig. 1 and Table 2.

### Implications for evolutionary and population genetics

A natural question arising from our results: what do these findings imply for evolutionary dynamics? Our analysis demonstrates that seascape features change appreciably across environments, but the *patterns* of change are system and scale-specific rather than universal. This has two key implications. First, it suggests that predictions about adaptation in fluctuating environments cannot rely on generic models of how landscapes change; instead, the specific structure of each seascape, including which scales of granularity are most sensitive to environmental perturbation, must be characterized empirically. Second, the observation that different seascapes respond differently to environmental variation implies that the evolutionary consequences of environmental fluctuations (e.g. whether fluctuations promote or constrain adaptation) will depend on the particular biological system. This motivates continued empirical characterization of seascapes rather than abandoning the enterprise, while also highlighting the need for theoretical frameworks that can accommodate this system-specificity.

Our findings demonstrate that environmental context appreciably alters all scales of seascape granularity. The variance decomposition analysis ($\eta_{\mathrm{env}}^{2}$, Supplementary Fig. S9) provides an initial quantitative comparison of how environmental variation partitions across scales. In the seascapes examined here, two patterns emerge. First, the allele scale consistently shows the highest $\eta_{\mathrm{env}}^{2}$, reflecting how the magnitude of genotype performance is highly dependent on the environment. Second, the locus-interaction scale consistently shows the lowest $\eta_{\mathrm{env}}^{2}$, with the environmental effect failing to reach significance in 11 of 12 seascapes. Because the interaction scale has a relatively large number of observations ($2^{L}$ coefficients per environment), this nonsignificance is unlikely to be solely a power issue, and suggests that the structure of epistatic interactions is relatively conserved across environments even when individual fitness values change substantially. This is not inconsistent with the MuRN analysis showing that individual $\gamma_{i}$ coefficients can change sign across environments. Rather, it indicates that such changes, while biologically meaningful, account for a small fraction of the total variance in epistatic coefficients compared to the variation across interactions within a single environment. We emphasize that these patterns describe the specific seascapes in our dataset and should not be generalized without further study. Different seascapes, particularly those involving larger genotype spaces or more extreme environmental perturbations, may show different patterns. The cross-scale comparison is also complicated by the fact that the statistics at each scale are nonlinear transformations of the same underlying fitness data (see caveats in Results). Nonetheless, the consistency of these patterns across a diverse set of biological systems is suggestive and motivates further investigation.

In interpreting the specific results of our analyses, several features are notable. The banal observation that allele performance changes across environments is not surprising, and the reaction norm highlights profound amounts of G × E interactions in both the pyrimethamine DHFR and LTEE datasets. This alone demonstrates the importance of the incorporation of environmental information in our descriptions of fitness landscape topography. But a more focused analysis of environmental epistasis and the shape of trajectories highlights the ways that environmental context dictates the pace and shape of adaptive evolution. The mutation-effect reaction norm (MuRN) (Figs. 4 and 5; Supplementary Figs. S3 and S4) demonstrates the prevalence of G × G × E interactions, where epistatic effects (themselves nonlinear and challenging to predict, by definition) change appreciably across environments (Ogbunugafor 2022). For example, in the LTEE seascape, the pairwise epistatic interaction between the 2nd and 3rd loci (*11**) has a negative $\gamma_{i}$ in both DM25 and DM25 + guanazole environments, but has a positive $\gamma_{i}$ in the DM25 + EGTA environment (Fig. 5a). This demonstrates that epistatic interactions manifest differently in different environments, with implications for the shape of trajectories and landscape topography. An important question is whether the observed changes in epistasis are a trivial consequence of changes in the rank order of fitness values across environments. While changes in rank order do necessarily entail some change in epistatic coefficients (since epistasis is derived from fitness values), the converse is not true: epistatic coefficients can change in sign and magnitude even when rank orders are preserved. In our data, we observe cases where epistatic coefficients change substantially even between environments with highly correlated rank orders (e.g. across closely spaced pyrimethamine concentrations in the DHFR seascape), suggesting that the changes in epistasis reflect genuine shifts in the structure of genotype-phenotype interactions beyond what is captured by rank-order changes alone. Likewise, when comparing how $C_{w(\text{avg})}$ changes between the DM25 + EGTA and DM25 + guanazole environments (Fig. 7c and d), we can infer that populations undergoing adaptive evolution can experience different extents of within-path interference on average, which can have implications for the speed of adaptive evolution across environments (Ogbunugafor and Eppstein 2016).

Our analysis across scales of granularity is useful because scientists or practitioners might be interested in the fitness seascape metaphor for different questions. For example, those interested in evolvability can focus on adaptive trajectories and their features (Wagner 2023; Ferrare and Good 2024; Jiménez-Sánchez et al. 2026). From our results, we can responsibly conclude that the fitness seascape metaphor provides enough variation across contexts to support its use as a meaningful advance on the fitness landscape, and a useful, (relatively) novel metaphor in population and evolutionary genetics.

### Implications for biomedicine and bioengineering

A disproportionate number of studies of empirical fitness landscapes have been conducted in disease systems, specifically those involving antimicrobial resistance (Lozovsky et al. 2009; Tan et al. 2011; Jiang et al. 2013; Mira et al. 2015; Weinreich et al. 2018; Guerrero et al. 2019; Ogbunugafor 2022; Kosterlitz et al. 2023). There are multiple reasons for this, including the availability of datasets, which relate to the public health interest in understanding and predicting the evolution of resistance (King et al. 2025). The seascape dimension further supports the notion that we can predict, control, or steer adaptive evolution. Since drug resistance arises in fluctuating environments, the emergence of the fitness seascape is an essential model for understanding how microbes evolve resistance to small molecules.

Another area where fitness landscapes have been increasingly discussed is bioengineering. The idea here is that the goal of engineering genomes requires knowledge of the underlying fitness landscape, as this can dictate how engineered mutations function with respect to genomic background. The fitness seascape metaphor also aids our perspective. For example, engineering a hypothetical crop with mutations conferring productivity through drought or disease conditions is not a static landscape problem – how the engineered mutations function across environmental contexts is central to whether it will be effective in real-world settings (Mittler and Blumwald 2010; Han et al. 2025).

Our $\eta_{\mathrm{env}}^{2}$ analysis adds a quantitative dimension to these considerations. For example, in a number of antimicrobial resistance seascapes, the trajectory scale is environmentally sensitive; which adaptive paths are accessible, and how quickly populations can traverse them, shifts dramatically with drug concentration, type, or even host species. This has direct clinical relevance. It suggests that the most consequential effect of changing drug environments may not be to alter the average fitness effects of individual resistance mutations (which are relatively stable across concentrations) but rather to reshape which evolutionary routes are available to a pathogen population. Two drug concentrations that produce similar rank orderings of genotype fitness can nonetheless differ substantially in which trajectories to resistance are accessible, implying that treatment protocols designed around static landscape assumptions may underestimate the diversity of evolutionary responses.

### Limitations

Although this study includes a multi-scale analysis of different fitness landscapes, what we have offered does not qualify as a comprehensive treatment of the ways that environmental-dependence can influence features of a fitness landscape. In Supplementary Information file S1, we describe additional metrics, including the edge flip fraction and global epistasis × environment interactions, that are germane to the study of fitness seascapes but were not computed in the present study. Nevertheless, we invite others to examine fitness seascapes using whatever approaches might be relevant to their area of interest.

We also note that our trajectory-scale metric $C_{w}$ captures only the sweep-time component of adaptive traversal and does not account for the waiting time for mutations to appear and establish in the population, which would be needed for a complete model of adaptation speed along a given path. Nonetheless, $C_{w}$ provides a useful comparative measure of how within-path competitive dynamics differ across environments.

In addition, our study focused on relatively small, combinatorially complete fitness landscapes. Although they are a foundational system in the study of empirical fitness landscapes, new technology has greatly increased the size of fitness landscapes that can be constructed and analyzed (Fowler and Fields 2014; Sarkisyan et al. 2016; Wu et al. 2016; Diss and Lehner 2018; Starr et al. 2022; Papkou et al. 2023; Johnston et al. 2024). Consequently, one may suggest that our proposed framework should be applied to larger datasets. Without digressing into a philosophical debate about the merits of small data versus big data, we offer the subtle claim that early in the rise of a concept (like the fitness seascape), using smaller, well-studied systems can be very useful, as the data have been vetted. Of course, the dichotomy between large and small data is not relegated to questions in evolutionary and population genetics, but is a larger issue that exists across many subfields of biology (and science) (Leonelli 2014; Todman et al. 2023).

### Conclusions

Our study contributes to a chorus aiming to fortify the fitness seascape as a valuable advance in the fields of evolutionary and population genetics. We provide a quantitative, multi-scale disentangling of context-dependence and environmental dynamism influencing molecular evolution. This has applications of various kinds, to evolutionary theorists, to clinicians who diagnose and treat diseases, and to biological engineers aiming to control how living systems function.

## Supplementary Material

jkag166_Supplementary_Data

## Data Availability

All code and data files are available at https://github.com/OgPlexus/Seascape1. Supplementary File S1 contains detailed descriptions of additional metrics as well as supplementary figures of analyses performed on two other seascapes that were referenced in the main text. Supplemental material available at G3 online.

## References

[jkag166-B1] Abreu CI, Mathur S, Petrov DA. 2024. Environmental memory alters the fitness effects of adaptive mutations in fluctuating environments. Nat Ecol Evol. 8:1760–1775. 10.1038/s41559-024-02475-9. 39020024 PMC11853131

[jkag166-B2] Agarwala A, Fisher DS. 2019. Adaptive walks on high-dimensional fitness landscapes and seascapes with distance-dependent statistics. Theor Popul Biol. 130:13–49. 10.1016/j.tpb.2019.09.011. 31605706

[jkag166-B3] Agur Z, Slobodkin L. 1986. Environmental fluctuations: how do they affect the topography of the adaptive landscape? J Genet. 65:45–54. 10.1007/BF02923535.

[jkag166-B4] Aita T, Iwakura M, Husimi Y. 2001. A cross-section of the fitness landscape of dihydrofolate reductase. Protein Eng. 14:633–638. 10.1093/protein/14.9.633. 11707608

[jkag166-B5] Ascensao JA, Desai MM. 2025. Experimental evolution in an era of molecular manipulation. Nat Rev Genet 27:81–95. 10.1038/s41576-024-00783-1. 40691351

[jkag166-B6] Bank C . 2022. Epistasis and adaptation on fitness landscapes. Annu Rev Ecol Evol Syst. 53:457–479. 10.1146/annurev-ecolsys-102320-112153.

[jkag166-B7] Brown KM et al 2010. Compensatory mutations restore fitness during the evolution of dihydrofolate reductase. Mol Biol Evol. 27:2682–2690. 10.1093/molbev/msq160. 20576759 PMC2981517

[jkag166-B8] Cairns J, Borse F, Mononen T, Hiltunen T, Mustonen V. 2022. Strong selective environments determine evolutionary outcome in time-dependent fitness seascapes. Evol Lett. 6:266–279. 10.1002/evl3.284. 35784450 PMC9233173

[jkag166-B9] Carja O, Liberman U, Feldman MW. 2014. Evolution in changing environments: modifiers of mutation, recombination, and migration. Proc Natl Acad Sci USA. 111:17935–17940. 10.1073/pnas.1417664111. 25427794 PMC4273399

[jkag166-B10] Cheetham T . 1993. Adaptation on fluctuating fitness landscapes: speciation and the persistence of lineages. J Theor Biol. 161:287–297. 10.1006/jtbi.1993.1056. 8331955

[jkag166-B11] Chen J, Fowler D, Tokuriki N. 2022. Environmental selection and epistasis in an empirical phenotype–environment–fitness landscape. Nat Ecol Evol. 6:427–438. 10.1038/s41559-022-01675-5. 35210579

[jkag166-B12] Chen P et al 2024. Inverted topographies in sequential fitness landscapes enable evolutionary control [preprint]. bioRxiv 630702. 10.1101/2024.12.29.630702.

[jkag166-B13] Cvijović I, Good BH, Jerison ER, Desai MM. 2015. Fate of a mutation in a fluctuating environment. Proc Natl Acad Sci USA. 112:E5021–E5028. 10.1073/pnas.1505406112. 26305937 PMC4568713

[jkag166-B14] da Silva J, Coetzer M, Nedellec R, Pastore C, Mosier DE. 2010. Fitness epistasis and constraints on adaptation in a human immunodeficiency virus type 1 protein region. Genetics. 185:293–303. 10.1534/genetics.109.112458. 20157005 PMC2870964

[jkag166-B15] Desponds J, Mora T, Walczak AM. 2016. Fluctuating fitness shapes the clone-size distribution of immune repertoires. Proc Natl Acad Sci USA. 113:274–279. 10.1073/pnas.1512977112. 26711994 PMC4720353

[jkag166-B16] De Visser JAG, Krug J. 2014. Empirical fitness landscapes and the predictability of evolution. Nat Rev Genet. 15:480–490. 10.1038/nrg3744. 24913663

[jkag166-B17] De Visser JAG, Park SC, Krug J. 2009. Exploring the effect of sex on empirical fitness landscapes. Am Nat. 174:S15–S30. 10.1086/599081. 19456267

[jkag166-B18] Diaz-Colunga J, Sanchez A, Ogbunugafor CB. 2023. Environmental modulation of global epistasis in a drug resistance fitness landscape. Nat Commun. 14:8055. 10.1038/s41467-023-43806-x. 38052815 PMC10698197

[jkag166-B19] Diss G, Lehner B. 2018. The genetic landscape of a physical interaction. Elife. 7:e32472. 10.7554/eLife.32472. 29638215 PMC5896888

[jkag166-B20] Doro S, Herman MA. 2022. On the fourier transform of a quantitative trait: implications for compressive sensing. J Theor Biol. 540:110985. 10.1016/j.jtbi.2021.110985. 34953868

[jkag166-B21] Faure AJ, Lehner B, Pina VM, Colome CS, Weghorn D. 2024. An extension of the Walsh-Hadamard transform to calculate and model epistasis in genetic landscapes of arbitrary shape and complexity. PLoS Comput Biol. 20:e1012132. 10.1371/journal.pcbi.1012132. 38805561 PMC11161127

[jkag166-B22] Ferrare JT, Good BH. 2024. Evolution of evolvability in rapidly adapting populations. Nat Ecol Evol. 8:2085–2096. 10.1038/s41559-024-02527-0. 39261599 PMC12049861

[jkag166-B23] Fisher RA . 1930. The genetical theory of natural selection. Clarendon Press.

[jkag166-B24] Flynn KM, Cooper TF, Moore FB, Cooper VS. 2013. The environment affects epistatic interactions to alter the topology of an empirical fitness landscape. PLoS Genet. 9:e1003426. 10.1371/journal.pgen.1003426. 23593024 PMC3616912

[jkag166-B25] Fowler DM, Fields S. 2014. Deep mutational scanning: a new style of protein science. Nat Methods. 11:801–807. 10.1038/nmeth.3027. 25075907 PMC4410700

[jkag166-B26] Fragata I, Blanckaert A, Louro MAD, Liberles DA, Bank C. 2019. Evolution in the light of fitness landscape theory. Trends Ecol Evol. 34:69–82. 10.1016/j.tree.2018.10.009. 30583805

[jkag166-B27] Gavrilets S . 2004. Fitness landscapes and the origin of species. Vol. 41. Princeton University Press.

[jkag166-B28] Gavrilets S . 2010. High-dimensional fitness landscapes and speciation. In: Evolution: The Extended Synthesis. The MIT Press.

[jkag166-B29] Gomulkiewicz R, Kirkpatrick M. 1992. Quantitative genetics and the evolution of reaction norms. Evolution. 46:390–411. 10.1111/j.1558-5646.1992.tb02047.x. 28564020

[jkag166-B30] Grefenstette JJ . 1999. Evolvability in dynamic fitness landscapes: a genetic algorithm approach. In: Proceedings of the 1999 Congress on Evolutionary Computation-CEC99 (Cat. No. 99TH8406). Vol. 3. p. 2031–2038.

[jkag166-B31] Guerrero RF, Dorji T, Ra’Mal MH, Shoulders MD, Ogbunugafor CB. 2024. Evolutionary druggability for low-dimensional fitness landscapes toward new metrics for antimicrobial applications. Elife. 12:RP88480. 10.7554/eLife.88480. 38833384 PMC11149929

[jkag166-B32] Guerrero RF, Scarpino SV, Rodrigues JV, Hartl DL, Ogbunugafor CB. 2019. Proteostasis environment shapes higher-order epistasis operating on antibiotic resistance. Genetics. 212:565–575. 10.1534/genetics.119.302138. 31015194 PMC6553834

[jkag166-B33] Hall AE et al 2019. Environment changes epistasis to alter trade-offs along alternative evolutionary paths. Evolution. 73:2094–2105. 10.1111/evo.13825. 31418459

[jkag166-B34] Hall DW, Agan M, Pope SC. 2010. Fitness epistasis among 6 biosynthetic loci in the budding yeast saccharomyces cerevisiae. J Heredity. 101:S75–S84. 10.1093/jhered/esq007. 20194517

[jkag166-B35] Han X et al 2025. et al Genetic engineering, including genome editing, for enhancing broad-spectrum disease resistance in crops. Plant Commun. 6:101195. 10.1016/j.xplc.2024.101195. PMC11897464 39568207

[jkag166-B36] Han X, Hui C. 2014. Niche construction on environmental gradients: the formation of fitness valley and stratified genotypic distributions. PLoS One. 9:e99775. 10.1371/journal.pone.0099775. 24915290 PMC4051751

[jkag166-B37] Hartl DL . 2014. What can we learn from fitness landscapes? Curr Opin Microbiol. 21:51–57. 10.1016/j.mib.2014.08.001. 25444121 PMC4254422

[jkag166-B38] Jiang PP, Corbett-Detig RB, Hartl DL, Lozovsky ER. 2013. Accessible mutational trajectories for the evolution of pyrimethamine resistance in the malaria parasite plasmodium vivax. J Mol Evol. 77:81–91. 10.1007/s00239-013-9582-z. 24071997

[jkag166-B39] Jiménez-Sánchez J, Ortega-Sabater C, Maini PK, Pérez-García VM, Lorenzi T. 2026. First explore, then settle: a theoretical analysis of evolvability as a driver of adaptation. Bull Math Biol. 88:21. 10.1007/s11538-025-01561-8. 41533262

[jkag166-B40] Johnston KE et al 2024. A combinatorially complete epistatic fitness landscape in an enzyme active site. Proc Natl Acad Sci USA. 121:e2400439121. 10.1073/pnas.2400439121. 39074291 PMC11317637

[jkag166-B41] Khan AI, Dinh DM, Schneider D, Lenski RE, Cooper TF. 2011. Negative epistasis between beneficial mutations in an evolving bacterial population. Science. 332:1193–1196. 10.1126/science.1203801. 21636772

[jkag166-B42] King E et al 2025. Fitness seascapes are necessary for realistic modeling of the evolutionary response to drug therapy. Sci Adv. 11, eadv1268. 10.1126/sciadv.adv1268. PMC12153978 40498821

[jkag166-B43] Kosterlitz O et al 2023. Evolutionary ‘crowdsourcing’: alignment of fitness landscapes allows for cross-species adaptation of a horizontally transferred gene. Mol Biol Evol. 40:msad237. 10.1093/molbev/msad237. 37931146 PMC10657783

[jkag166-B44] Kryazhimskiy S, Rice DP, Jerison ER, Desai MM. 2014. Global epistasis makes adaptation predictable despite sequence-level stochasticity. Science. 344:1519–1522. 10.1126/science.1250939. 24970088 PMC4314286

[jkag166-B45] Kryazhimskiy S, Tkačik G, Plotkin JB. 2009. The dynamics of adaptation on correlated fitness landscapes. Proc Natl Acad Sci USA. 106:18638–18643. 10.1073/pnas.0905497106. 19858497 PMC2767361

[jkag166-B46] Kumawat B, Lalejini A, Acosta MM, Zaman L. 2025. Evolution takes multiple paths to evolvability when facing environmental change. Proc Natl Acad Sci USA. 122:e2413930121. 10.1073/pnas.2413930121. 39739809 PMC11725885

[jkag166-B47] Lande R . 1976. Natural selection and random genetic drift in phenotypic evolution. Evolution. 30:314–334. 10.1111/j.1558-5646.1976.tb00911.x. 28563044

[jkag166-B48] Lässig M, Mustonen V, Walczak AM. 2017. Predicting evolution. Nat Ecol Evol. 1:0077. 10.1038/s41559-017-0077. 28812721

[jkag166-B49] Leonelli S . 2014. What difference does quantity make? On the epistemology of big data in biology. Big Data Soc. 1:2053951714534395. 10.1177/2053951714534395. PMC4340542 25729586

[jkag166-B50] Li C, Zhang J. 2018. Multi-environment fitness landscapes of a tRNA gene. Nat Ecol Evol. 2:1025–1032. 10.1038/s41559-018-0549-8. 29686238 PMC5966336

[jkag166-B51] Lindsey HA, Gallie J, Taylor S, Kerr B. 2013. Evolutionary rescue from extinction is contingent on a lower rate of environmental change. Nature. 494:463–467. 10.1038/nature11879. 23395960

[jkag166-B52] Lozovsky ER et al 2009. Stepwise acquisition of pyrimethamine resistance in the malaria parasite. Proc Natl Acad Sci USA. 106:12025–12030. 10.1073/pnas.0905922106. 19587242 PMC2715478

[jkag166-B53] Maltas J, McNally DM, Wood KB. 2021. Evolution in alternating environments with tunable interlandscape correlations. Evolution. 75:10–24. 10.1111/evo.14121. 33206376 PMC8246403

[jkag166-B54] Martin G, Elena SF, Lenormand T. 2007. Distributions of epistasis in microbes fit predictions from a fitness landscape model. Nat Genet. 39:555–560. 10.1038/ng1998. 17369829

[jkag166-B55] Martin G, Lenormand T. 2006. The fitness effect of mutations across environments: a survey in light of fitness landscape models. Evolution. 60:2413–2427. 10.1111/j.0014-3820.2006.tb01878.x. 17263105

[jkag166-B56] Merrell DJ . 1994. The adaptive seascape: the mechanism of evolution. U of Minnesota Press.

[jkag166-B57] Mira PM, Meza JC, Nandipati A, Barlow M. 2015. Adaptive landscapes of resistance genes change as antibiotic concentrations change. Mol Biol Evol. 32:2707–2715. 10.1093/molbev/msv146. 26113371

[jkag166-B58] Mittler R, Blumwald E. 2010. Genetic engineering for modern agriculture: challenges and perspectives. Annu Rev Plant Biol. 61:443–462. 10.1146/annurev-arplant-042809-112116. 20192746

[jkag166-B59] Mustonen V, Lässig M. 2009. From fitness landscapes to seascapes: non-equilibrium dynamics of selection and adaptation. Trends Genet. 25:111–119. 10.1016/j.tig.2009.01.002. 19232770

[jkag166-B60] Ogbunugafor CB . 2022. The mutation effect reaction norm (mu-rn) highlights environmentally dependent mutation effects and epistatic interactions. Evolution. 76:37–48. 10.1111/evo.14428. 34989399

[jkag166-B61] Ogbunugafor CB, Eppstein MJ. 2016. Competition along trajectories governs adaptation rates towards antimicrobial resistance. Nat Ecol Evol. 1:0007. 10.1038/s41559-016-0007. 28812552

[jkag166-B62] Ogbunugafor CB, Hartl D. 2016. A pivot mutation impedes reverse evolution across an adaptive landscape for drug resistance in plasmodium vivax. Malar J. 15:40. 10.1186/s12936-016-1090-3. 26809718 PMC4727274

[jkag166-B63] Ogbunugafor CB, Wylie CS, Diakite I, Weinreich DM, Hartl DL. 2016. Adaptive landscape by environment interactions dictate evolutionary dynamics in models of drug resistance. PLoS Comput Biol. 12:e1004710. 10.1371/journal.pcbi.1004710. 26808374 PMC4726534

[jkag166-B64] O’Maille PE et al 2008. Quantitative exploration of the catalytic landscape separating divergent plant sesquiterpene synthases. Nat Chem Biol. 4:617–623. 10.1038/nchembio.113. 18776889 PMC2664519

[jkag166-B65] Oomen RA, Hutchings JA. 2015. Genetic variability in reaction norms in fishes. Environ Rev. 23:353–366. 10.1139/er-2014-0077.

[jkag166-B66] Oomen RA, Kuparinen A, Hutchings JA. 2020. Consequences of single-locus and tightly linked genomic architectures for evolutionary responses to environmental change. J Heredity. 111:319–332. 10.1093/jhered/esaa020. PMC7423069 32620014

[jkag166-B67] Papkou A, Garcia-Pastor L, Escudero JA, Wagner A. 2023. A rugged yet easily navigable fitness landscape. Science. 382:eadh3860. 10.1126/science.adh3860. 37995212

[jkag166-B68] Peacor SD, Allesina S, Riolo RL, Pascual M. 2006. Phenotypic plasticity opposes species invasions by altering fitness surface. PLoS Biol. 4:e372. 10.1371/journal.pbio.0040372. 17076585 PMC1629039

[jkag166-B69] Petak C, Frati L, Vroomans RM, Pespeni MH, Cheney N. 2025. The variability of evolvability: properties of dynamic fitness landscapes determine how phenotypic variability evolves. Proc Natl Acad Sci USA. 122:e2519469122. 10.1073/pnas.2519469122. 41397131 PMC12745803

[jkag166-B70] Pitzer E, Affenzeller M. 2012. A comprehensive survey on fitness landscape analysis. In: Fodor, J., Klempous, R., Suárez Araujo, C.P. (eds) Recent Advances in Intelligent Engineering Systems. Studies in Computational Intelligence. Vol. 378. p. 161–191. Springer, Berlin, Heidelberg. 10.1007/978-3-642-23229-9_8

[jkag166-B71] Poelwijk FJ, Krishna V, Ranganathan R. 2016. The context-dependence of mutations: a linkage of formalisms. PLoS Comput Biol. 12:e1004771. 10.1371/journal.pcbi.1004771. 27337695 PMC4919011

[jkag166-B72] Rodrigues J et al 2016. Biophysical principles predict fitness landscapes of drug resistance. Proc Natl Acad Sci USA. 113:E1470–E1478. 10.1073/pnas.1601441113. 26929328 PMC4801265

[jkag166-B73] Rozen DE, Habets MG, Handel A, de Visser JAG. 2008. Heterogeneous adaptive trajectories of small populations on complex fitness landscapes. PLoS One. 3:e1715. 10.1371/journal.pone.0001715. 18320036 PMC2248617

[jkag166-B74] Saakian DB, Yakushkina T, Koonin EV. 2019. Allele fixation probability in a Moran model with fluctuating fitness landscapes. Phys Rev E. 99:022407. 10.1103/PhysRevE.99.022407. 30934266

[jkag166-B75] Sae-Lim P, Gjerde B, Nielsen HM, Mulder H, Kause A. 2016. A review of genotype-by-environment interaction and micro-environmental sensitivity in aquaculture species. Rev Aquac. 8:369–393. 10.1111/raq.12098.

[jkag166-B76] Sarkisyan KS et al 2016. Local fitness landscape of the green fluorescent protein. Nature. 533:397–401. 10.1038/nature17995. 27193686 PMC4968632

[jkag166-B77] Schlichting CD, Smith H. 2002. Phenotypic plasticity: linking molecular mechanisms with evolutionary outcomes. Evol Ecol. 16:189–211. 10.1023/A:1019624425971.

[jkag166-B78] Smith T, Husbands P, O’Shea M. 2002. Fitness landscapes and evolvability. Evol Comput. 10:1–34. 10.1162/106365602317301754. 11911781

[jkag166-B79] Starr TN et al 2022. Shifting mutational constraints in the SARS-COV-2 receptor-binding domain during viral evolution. Science. 377:420–424. 10.1126/science.abo7896. 35762884 PMC9273037

[jkag166-B80] Szendro IG, Schenk MF, Franke J, Krug J, De Visser JAG. 2013. Quantitative analyses of empirical fitness landscapes. J Stat Mech. 2013:P01005. 10.1088/1742-5468/2013/01/P01005.

[jkag166-B81] Tan L, Serene S, Chao HX, Gore J. 2011. Hidden randomness between fitness landscapes limits reverse evolution. Phys Rev Lett. 106:198102. 10.1103/PhysRevLett.106.198102. 21668204

[jkag166-B82] Tanaka MM, Godfrey-Smith P, Kerr B. 2020. The dual landscape model of adaptation and niche construction. Philos Sci. 87:478–498. 10.1086/708692.

[jkag166-B83] Tenaillon O, Toupance B, Le Nagard H, Taddei F, Godelle B. 1999. Mutators, population size, adaptive landscape and the adaptation of asexual populations of bacteria. Genetics. 152:485–493. 10.1093/genetics/152.2.485. 10353893 PMC1460623

[jkag166-B84] Todman LC, Bush A, Hood AS. 2023. ‘small data’ for big insights in ecology. Trends Ecol Evol. 38:615–622. 10.1016/j.tree.2023.01.015. 36797167

[jkag166-B85] Trubenova B, Krejca MS, Lehre PK, Kötzing T. 2019. Surfing on the seascape: adaptation in a changing environment. Evolution. 73:1356–1374. 10.1111/evo.13784. 31206653 PMC6771940

[jkag166-B86] Wagner A . 2023. Evolvability-enhancing mutations in the fitness landscapes of an rna and a protein. Nat Commun. 14:3624. 10.1038/s41467-023-39321-8. 37336901 PMC10279741

[jkag166-B87] Watson RA, Weinreich DM, Wakeley J. 2011. Genome structure and the benefit of sex. Evolution. 65:523–536. 10.1111/j.1558-5646.2010.01144.x. 21029076

[jkag166-B88] Weinreich DM, Delaney NF, DePristo MA, Hartl DL. 2006. Darwinian evolution can follow only very few mutational paths to fitter proteins. Science. 312:111–114. 10.1126/science.1123539. 16601193

[jkag166-B89] Weinreich DM, Lan Y, Jaffe J, Heckendorn RB. 2018. The influence of higher-order epistasis on biological fitness landscape topography. J Stat Phys. 172:208–225. 10.1007/s10955-018-1975-3. 29904213 PMC5986866

[jkag166-B90] Weinreich DM, Lan Y, Wylie CS, Heckendorn RB. 2013. Should evolutionary geneticists worry about higher-order epistasis? Curr Opin Genet Dev. 23:700–707. 10.1016/j.gde.2013.10.007. 24290990 PMC4313208

[jkag166-B91] Wright S . 1932. The roles of mutation, inbreeding, crossbreeding, and selection in evolution. Proc Sixth Int Cong Genet. 1:356–366.

[jkag166-B92] Wu NC, Dai L, Olson CA, Lloyd-Smith JO, Sun R. 2016. Adaptation in protein fitness landscapes is facilitated by indirect paths. Elife. 5:e16965. 10.7554/eLife.16965. 27391790 PMC4985287

