# Supplementary material for: Deconstructing empirical fitness seascapes across scales of granularity
Source: G3 (Bethesda). 2026 Jun 26;16(9):jkag166. doi: 10.1093/g3journal/jkag166 (PMC13535436; doi:10.1093/g3journal/jkag166)
Supplement: Supplementary file 1 [file jkag166_supplementary_data.zip › Supplementary Material/ManivannanOgbunugafor_SupplementaryFileS1.pdf]

Supplementary Material

Deconstructing empirical fitness seascapes across scales of granularity

Swathi Nachiar Manivannan[1] and C. Brandon Ogbunugafor[1,2,*]

[1]Department of Ecology & Evolutionary Biology, Yale University, New Haven, CT, 06511, USA
[2]Public Health Modeling Unit, Yale School of Public Health, New Haven, CT, 06511 USA
*CBO: brandon.ogbunu@yale.edu

Contents of this supplementary material file:

S1 Additional approaches for studying fitness seascapes **2**
S1.1 Edge flip fraction 2
S1.2 Global epistasis × environment interactions 2

S2 Analyses at the locus scale for 2 seascapes **4**

S3 Analyses at the locus-interaction scale for 2 seascapes **6**

S4 Analyses at the allele scale for 2 seascapes **8**

S5 Analyses at the trajectory and landscape topography scales **9**

S6 Analysis of environmental sensitivity for all seascapes **12**

Notes on the Supplementary Material

In this manuscript, we propose a framework for deconstructing the study of fitness seascapes across scales of granularity, primarily focusing on two empirical seascapes. In the supplement, we present analyses of two additional empirical seascapes at different scales of granularity to provide a more comprehensive discussion of the framework. We also provide analyses of all 12 empirical seascapes mentioned in Table 1 (in the main text) at the landscape scale to offer general insights on how trends at the landscape scale have manifested across empirical seascapes.

S1 Additional approaches for studying fitness seascapes

Besides the metrics mentioned in the main text of our manuscript, several theoretical and empirical approaches to the study of evolution under fluctuating environments more generally have also been developed. While these approaches do not typically utilize the small, combinatorially complete seascape datasets we used in our framework, they nonetheless address central questions. Some have addressed how the fixation probability of a novel mutation is dependent on the environmental variability a population is subject to (***locus-scale analysis***) [*1*], how mutation rates can evolve in response to environmental and demographic fluctuations (***landscape scale-trajectory analysis***) [*2*], how fitness variance of mutations may affect 'environmental memory' and hence evolution across fluctuating environments (***locus-scale analysis***) [*3*], and how environmental variability can drive mutational robustness, evolvability, fitness distributions, and diversity of populations (***locus, landscape scale-trajectory analyses***) [*4*,*5*]. Here, we describe two additional metrics that could be incorporated into future studies of fitness seascapes.

S1.1 Edge flip fraction

The edge flip fraction is a landscape-scale metric that quantifies the shift in topography of a fitness landscape between two environmental conditions [*6*]. Briefly, it calculates the fraction of edge flips between alleles (nodes) for two environments, i.e., how many mutational steps between neighboring genotypes change from beneficial/deleterious to deleterious/beneficial. Computing the edge flip fraction, $\mathcal{E}$, involves constructing a transition matrix between nodes for two landscapes, A and B, to first determine the edge flip matrix, E:

$$\mathbf{E} = \frac{[(U^{\lceil \mathbf{A} \rceil} - L^{\lceil \mathbf{A} \rceil^T}) - (U^{\lceil \mathbf{B} \rceil} - L^{\lceil \mathbf{B} \rceil^T})]}{2}, \tag{1}$$

where $U^{\lceil \mathbf{X} \rceil}$ denotes the upper triangular portion of a matrix $\mathbf{X}$ with the ceiling function applied to each element, and $L^{\lceil \mathbf{X} \rceil^T}$ denotes the transposed lower triangular portion of a matrix $\mathbf{X}$ with the ceiling function applied to each element. $\mathcal{E}$ is then calculated as:

$$\mathcal{E} = \frac{\sum_{ij} \mid \mathbf{e}_{ij} \mid}{N \times 2^N/2}, \mathcal{E} = [0, 1], \tag{2}$$

where $\mathcal{E} = 0$ denotes no change in evolutionary direction between two environments and $\mathcal{E} = 1$ denotes change in evolutionary direction for all steps between the environments.

S1.2 Global epistasis × environment interactions

Another approach to analyzing how epistasis in adaptive landscapes is modulated by environmental context is to look at *global epistasis*, where the fitness effect of a mutation (Δr) can be predicted by the fitness of the genomic background where it is added [*7*–*9*].

To compute the global epistasis of a given mutation i, we can first compute the fitness effect of mutation i across the entire set of genetic backgrounds (i.e., the set of genotypes not carrying mutation i) for every background B in the fitness landscape,

$$\Delta r_i = r(B + i) - r(B), \tag{3}$$

where $r(B + i)$ denotes the fitness of genotype $B + i$ and $r(B)$ the fitness of genotype B (i.e., genetic background). From the fitness effect, we can then infer different characteristics about the epistasis for a given mutation, such as the directionality of epistasis (slope of linear regression between Δr and $r(B)$), the 'globality' of epistasis (R^2 of linear regression between Δr and $r(B)$), and the strength (magnitude) of epistasis ($\frac{\text{var}(\Delta r_i)}{\text{var}(r(B))}$). We can therefore adapt the environmental epistasis approach by considering how *global epistasis* is modulated by the environment for larger landscapes, which offers another way of analyzing how interactions between loci are shaped by the environment [10].

S2 Analyses at the locus scale for 2 seascapes

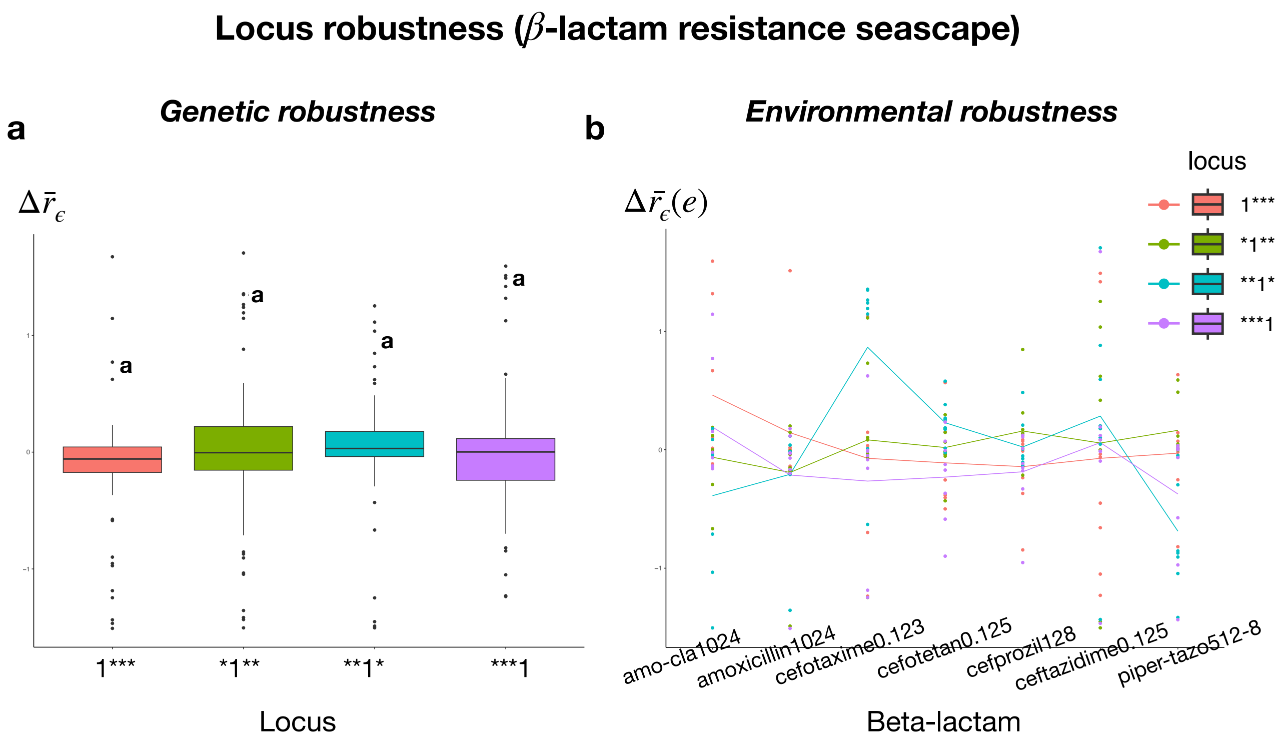

Fig. S1. Locus-scale analyses of genetic and environmental robustness for 2^4 bla$_{TEM}$ genotypes in *E. coli* across 7 different β-lactam antibiotics (amoxicillin + clavulanic acid (1024 μg ml^{-1}), amoxicillin (1024 μg ml^{-1}), cefotaxime (0.123 μg ml^{-1}), cefotetan (0.125 μg ml^{-1}), cefprozil (128 μg ml^{-1}), ceftazidime (0.125 μg ml^{-1}), piperacillin/tazobactam (512/8 μg ml^{-1})) in the β-lactam resistance seascape [*11*], where each locus ϵ is represented by a unique color. (a) shows a boxplot of $\Delta\bar{r}_\epsilon$ for each locus (***genetic robustness***, i.e., $r_{j\epsilon}(e) - r_{j0}(e)$ averaged across all genetic backgrounds and environments for every locus ϵ), with compact letter display of one-way ANOVA and Tukey's posthoc tests. (b) shows $\Delta\bar{r}_\epsilon(e)$ for loci averaged across all genetic backgrounds for each of the individual environmental conditions (***environmental robustness***) for each of the 7 β-lactam antibiotics. Each point represents $r_{j\epsilon}(e) - r_{j0}(e)$ for every combination of ϵ and e, and the lines represent mean trend lines for $\Delta\bar{r}_\epsilon(e)$ across all environments.

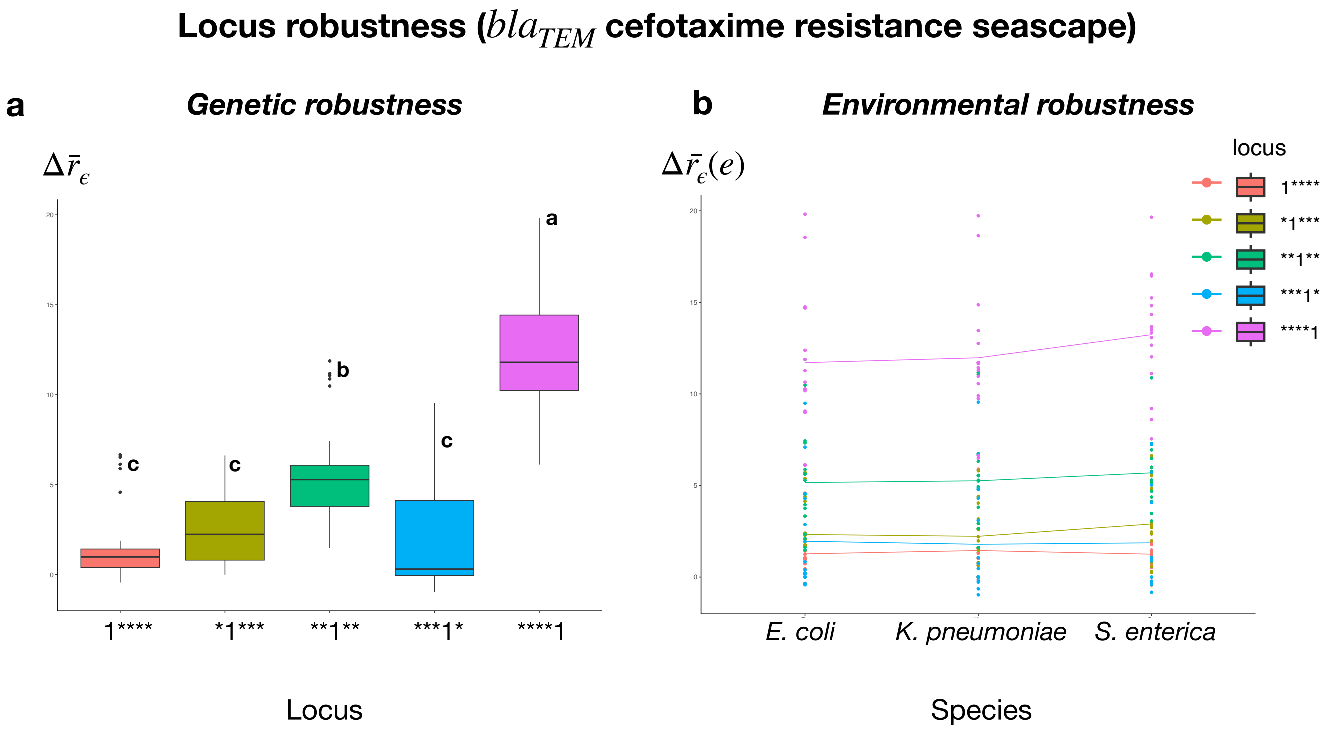

Fig. S2. Locus-scale analyses of genetic and environmental robustness for 2^5 bla$_{TEM}$ genotypes across 3 bacterial species in the bla$_{TEM}$ cefotaxime resistance seascape [12], where each locus ϵ is represented by a unique color. (a) shows a boxplot of $\Delta\bar{r}_\epsilon$ for each locus (***genetic robustness***, i.e., $r_{j\epsilon}(e) - r_{j0}(e)$ averaged across all genetic backgrounds and environments for every locus ϵ), with compact letter display of one-way ANOVA and Tukey's posthoc tests. (b) shows $\Delta\bar{r}_\epsilon(e)$ for loci averaged across all genetic backgrounds for each of the individual environmental conditions (***environmental robustness***) for each of the 3 bacterial species. Each point represents $r_{j\epsilon}(e) - r_{j0}(e)$ for every combination of ϵ and e, and the lines represent mean trend lines for $\Delta\bar{r}_\epsilon(e)$ across all environments.

S3 Analyses at the locus-interaction scale for 2 seascapes

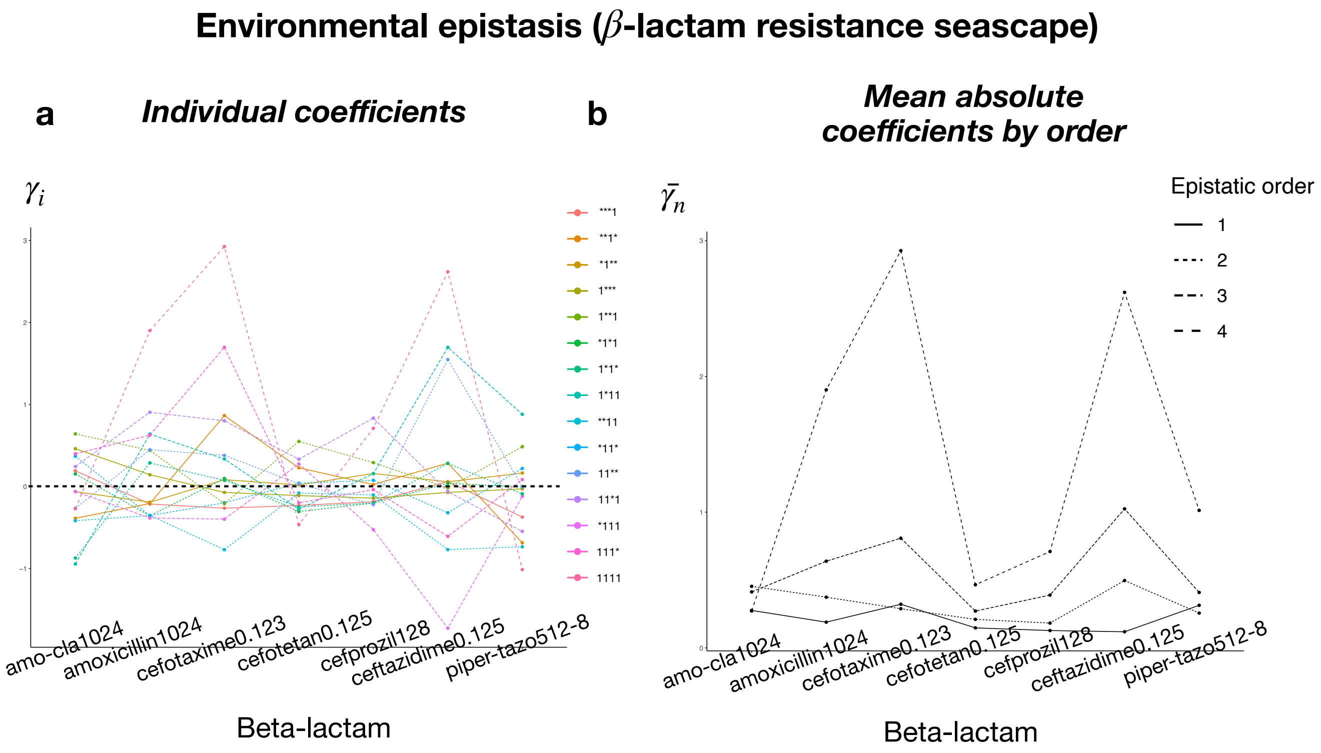

Fig. S3. Locus-interaction analyses of environmental epistasis, as computed through the mutation-effect reaction norm (MuRN), for 2^4 bla$_{TEM}$ genotypes in *E. coli* across 7 different β-lactam antibiotics in the β-lactam resistance seascape [*11*]. (a) shows the MuRN depicting coefficients of every possible epistatic interaction (γ_i) across all 7 antibiotics (with a black horizontal dashed line to indicate $\gamma_i = 0$), while (b) shows the absolute mean of epistatic coefficients based on epistatic order ($\bar{\gamma}_n$). Note that for all figures, the 0th-order interaction is not shown.

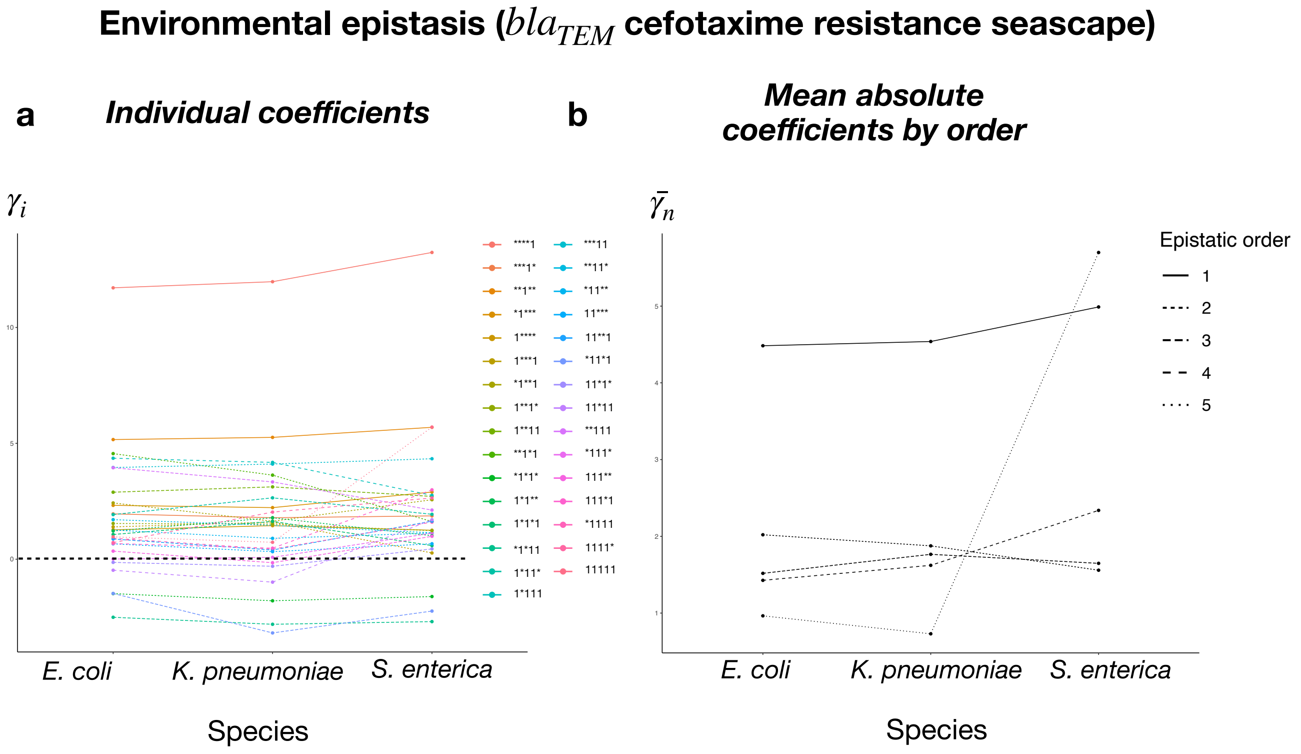

Fig. S4. Locus-interaction analyses of environmental epistasis, as computed through the mutation-effect reaction norm (MuRN), for 2^5 bla$_{TEM}$ genotypes across 3 bacterial species in the bla$_{TEM}$ cefotaxime resistance seascape [12]. (a) shows the MuRN depicting coefficients of every possible epistatic interaction (γ_i) across all three bacterial species (with a black horizontal dashed line to indicate $\gamma_i = 0$), while (b) shows the absolute mean of epistatic coefficients based on epistatic order ($\bar{\gamma}_n$). Note that for all figures, the 0th-order interaction is not shown.

S4 Analyses at the allele scale for 2 seascapes

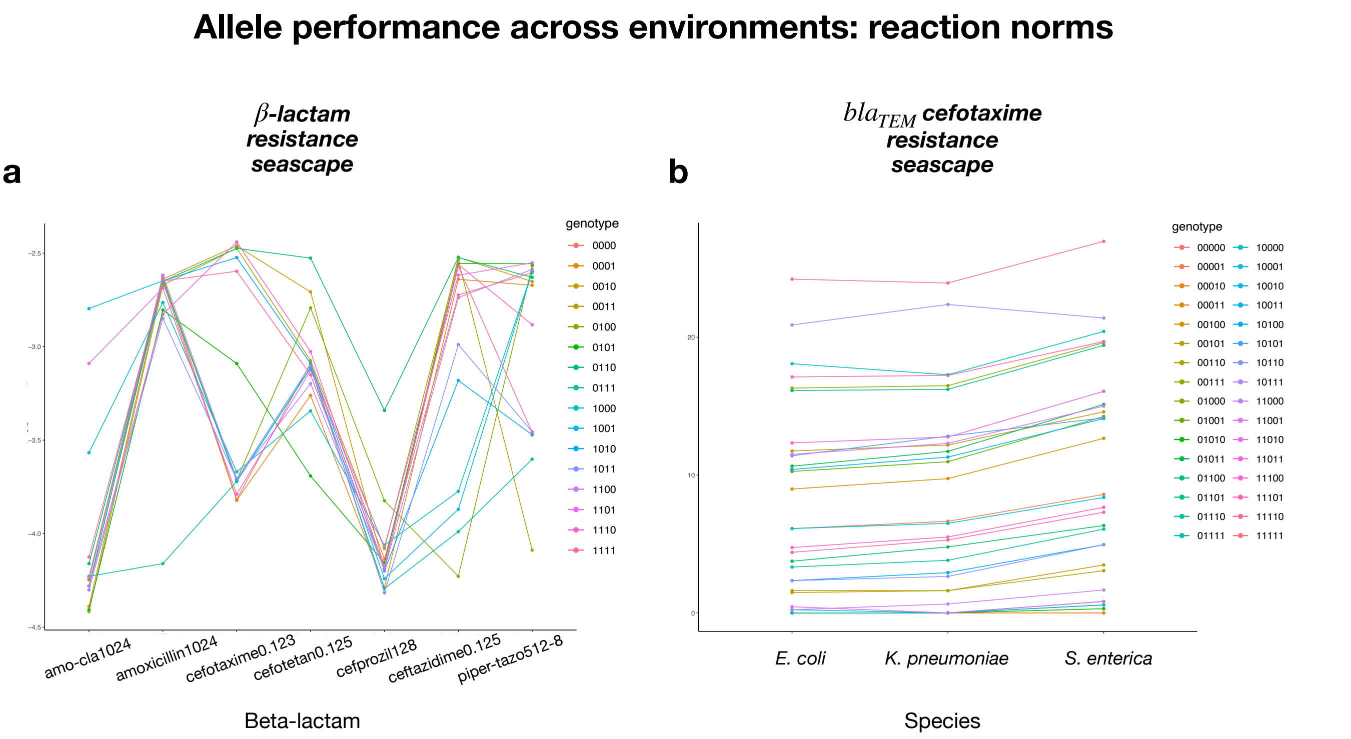

Fig. S5. Allele performance across environments: reaction norms. Genotype performance in every environment is represented by a differently colored line for each genotype. (a) Reaction norms of the β-lactam resistance seascape [11], demonstrating the growth rate of 2^4 bla$_{TEM}$ genotypes in *E. coli* across 7 different β-lactam antibiotics. (b) Reaction norms of the bla$_{TEM}$ cefotaxime resistance seascape [12], demonstrating the relative resistance level of 2^5 bla$_{TEM}$ genotypes against cefotaxime across 3 bacterial species.

S5 Analyses at the trajectory and landscape topography scales

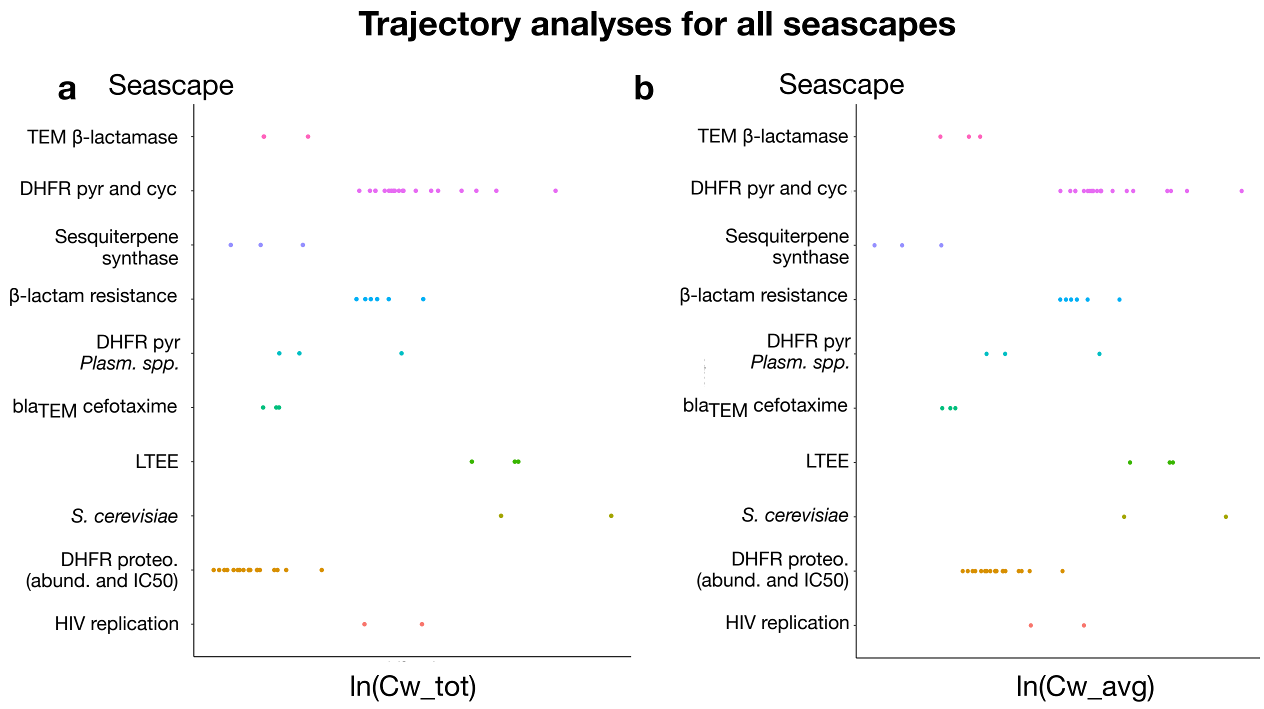

Fig. S6. Trajectory analyses: (a) Distribution of $C_{w(tot)}$ under different environmental and/or phenotypic contexts for all 12 seascapes. Note that DHFR pyrimethamine and DHFR cycloguanil seascapes are merged and DHFR proteostasis (IC50 and abundance) seascapes are merged in this figure. (b) Distribution of $C_{w(avg)}$ under different environmental and/or phenotypic contexts for all 12 seascapes. Note that DHFR pyrimethamine and DHFR cycloguanil seascapes are merged and DHFR proteostasis (IC50 and abundance) seascapes are merged in this figure.

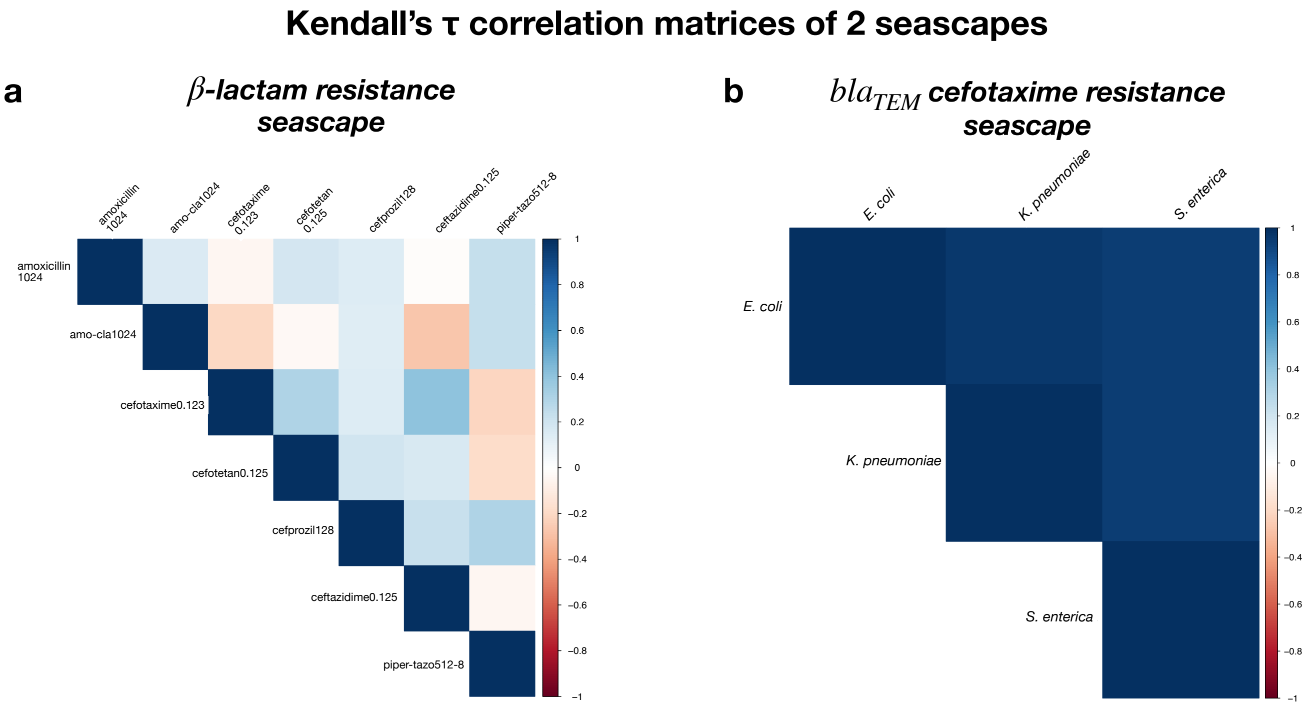

Fig. S7. Landscape-scale analyses: Kendall's τ correlation matrices of genotype rank orders for pairs of environments. (a) Kendall's τ correlation matrix for 7 different β-lactam antibiotics in the β-lactam resistance seascape [11], (b) Kendall's τ correlation matrix for 3 different bacterial species in the bla$_{TEM}$ cefotaxime resistance seascape [12].

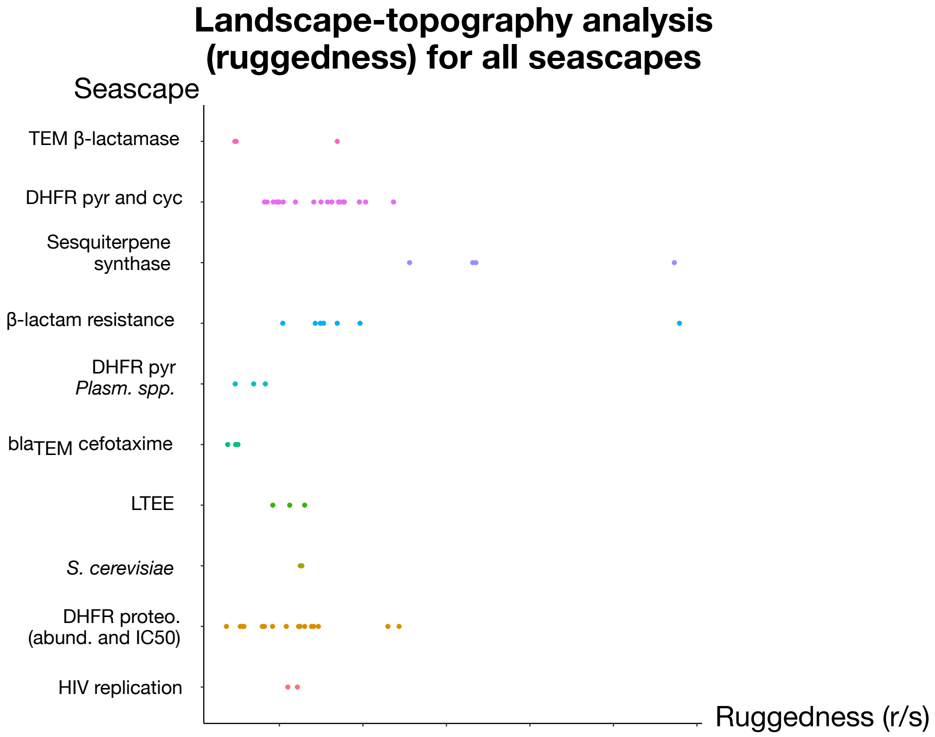

Fig. S8. Distribution of roughness-to-slope ratios under different environmental and/or phenotypic contexts for all 12 seascapes. Note that DHFR pyrimethamine and DHFR cycloguanil seascapes are merged and DHFR proteostasis (IC50 and abundance) seascapes are merged in this figure.

Table S1. Reference table for genotypes and their corresponding node numbers in Figure 8 a, b, d, e (in the main text).

Seascape	Allele	Corresponding node number	Hamming distance
DHFR pyrimethamine	0000	1	0
	0001	2	1
	0010	3	1
	0011	4	2
	0100	5	1
	0101	6	2
	0110	7	2
	0111	8	3
	1000	9	1
	1001	10	2
	1010	11	2
	1011	12	3
	1100	13	2
	1101	14	3
	1110	15	3
	1111	16	4
LTEE	00000	1	0
	00001	2	1
	00010	3	1
	00011	4	2
	00100	5	1
	00101	6	2
	00110	7	2
	00111	8	3
	01000	9	1
	01001	10	2
	01010	11	2
	01011	12	3
	01100	13	2
	01101	14	3
	01110	15	3
	01111	16	4
	10000	17	1
	10001	18	2
	10010	19	2
	10011	20	3
	10100	21	2
	10101	22	3
	10110	23	3
	10111	24	4
	11000	25	2
	11001	26	3
	11010	27	3
	11011	28	4
	11100	29	3
	11101	30	4
	11110	31	4
	11111	32	5

S6 Analysis of environmental sensitivity for all seascapes

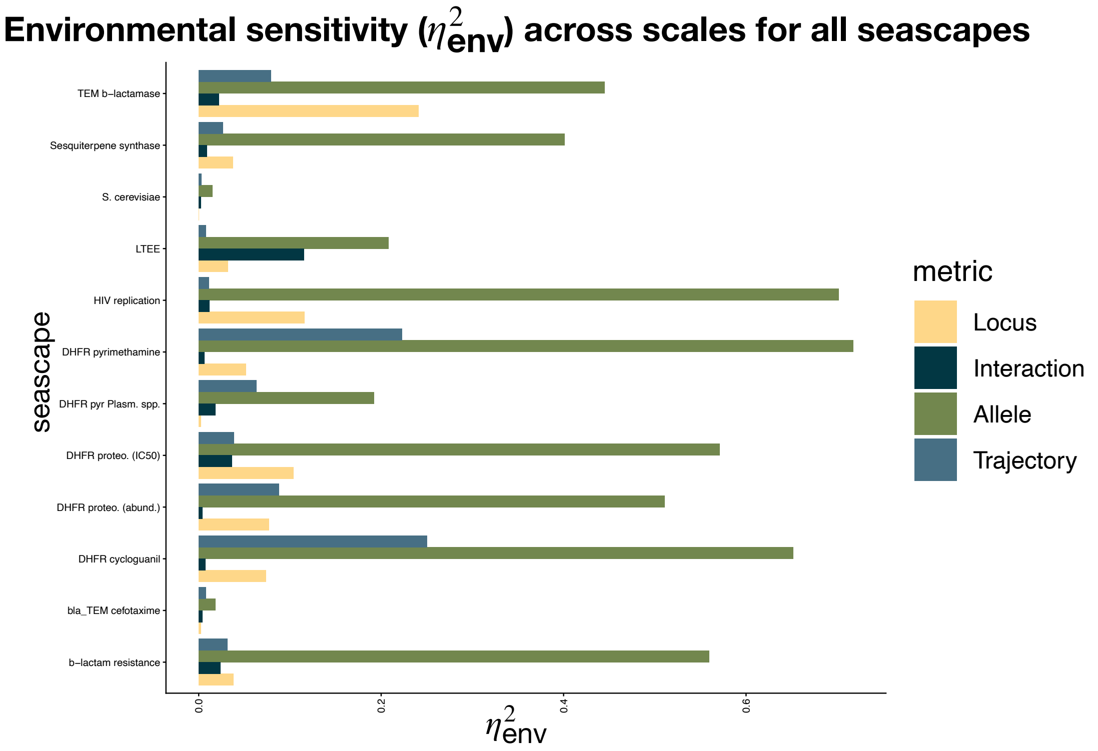

Fig. S9. Environmental sensitivity (η^2_{env}) across scales of granularity for all seascapes. Each group of bars represents one seascape; bar colors indicate the scale of analysis. The allele-scale consistently shows the highest environmental sensitivity, while the locus-interaction scale shows the lowest. See Table 3 in the main text for numerical values.

References

1. Cvijović I, Good BH, Jerison ER and Desai MM (2015). Fate of a mutation in a fluctuating environment. *Proceedings of the National Academy of Sciences* **112(36)**:E5021–E5028

2. Wei W, Ho WC, Behringer MG, Miller SF, Bcharah G and Lynch M (2022). Rapid evolution of mutation rate and spectrum in response to environmental and population-genetic challenges. *Nature communications* **13(1)**:4752

3. Abreu CI, Mathur S and Petrov DA (2024). Environmental memory alters the fitness effects of adaptive mutations in fluctuating environments. *Nature ecology & evolution* **8(9)**:1760–1775

4. Desponds J, Mora T and Walczak AM (2016). Fluctuating fitness shapes the clone-size distribution of immune repertoires. *Proceedings of the National Academy of Sciences* **113(2)**:274–279

5. Petak C, Frati L, Vroomans RM, Pespeni MH and Cheney N (2025). The variability of evolvability: Properties of dynamic fitness landscapes determine how phenotypic variability evolves. *Proceedings of the National Academy of Sciences* **122(51)**:e2519469122

6. Chen S, Rabenberg A, Bhatt S, Bhattacharyya RP, Ester E, Gowda K, Blainey PC and Hung DT (2024). Inverted fitness landscapes and the evolution of antibiotic resistance. *Nature Ecology & Evolution*

7. MacLean RC, Perron GG and Gardner A (2010). Diminishing returns from beneficial mutations and pervasive epistasis shape the fitness landscape for rifampicin resistance in *Pseudomonas aeruginosa*. *Genetics* **186(4)**:1345–1354

8. Diaz-Colunga J, Barrat-Charlaix P, Krug J and Liebermeister W (2023). Global epistasis on fitness landscapes. *Philosophical Transactions of the Royal Society B* **378(1877)**:20220053

9. Johnson MS, Martsul A, Kryazhimskiy S and Desai MM (2023). Epistasis and evolution: Recent advances and an outlook for prediction. *BMC Biology* **21(1)**:120

10. Diaz-Colunga J, Skwara A, Gowda K, Diez-Hermano S, Goberna-Ferrón S, Harcombe WR and Sanchez A (2023). Environmental modulation of global epistasis in a drug resistance fitness landscape. *Nature Communications* **14**:8055

11. Mira PM, Meza JC, Nandipati A and Barlow M (2015). Adaptive landscapes of resistance genes change as antibiotic concentrations change. *Molecular biology and evolution* **32(10)**:2707–2715

12. Kosterlitz O, Grassi N, Werner B, McGee RS, Top EM and Kerr B (2023). Evolutionary “crowdsourcing”: alignment of fitness landscapes allows for cross-species adaptation of a horizontally transferred gene. *Molecular Biology and Evolution* **40(11)**:msad237
