# Supplementary material for: Deconstructing empirical fitness seascapes across scales of granularity
Source: G3 (Bethesda). 2026 Jun 26;16(9):jkag166. doi: 10.1093/g3journal/jkag166 (PMC13535436; doi:10.1093/g3journal/jkag166)
Supplement: Supplementary file 1 [file jkag166_supplementary_data.zip › Supplementary Material/data_code/walsh_hadamard/khan2011flynn2013/murn_khan2011flynn2013_20251203.pdf]

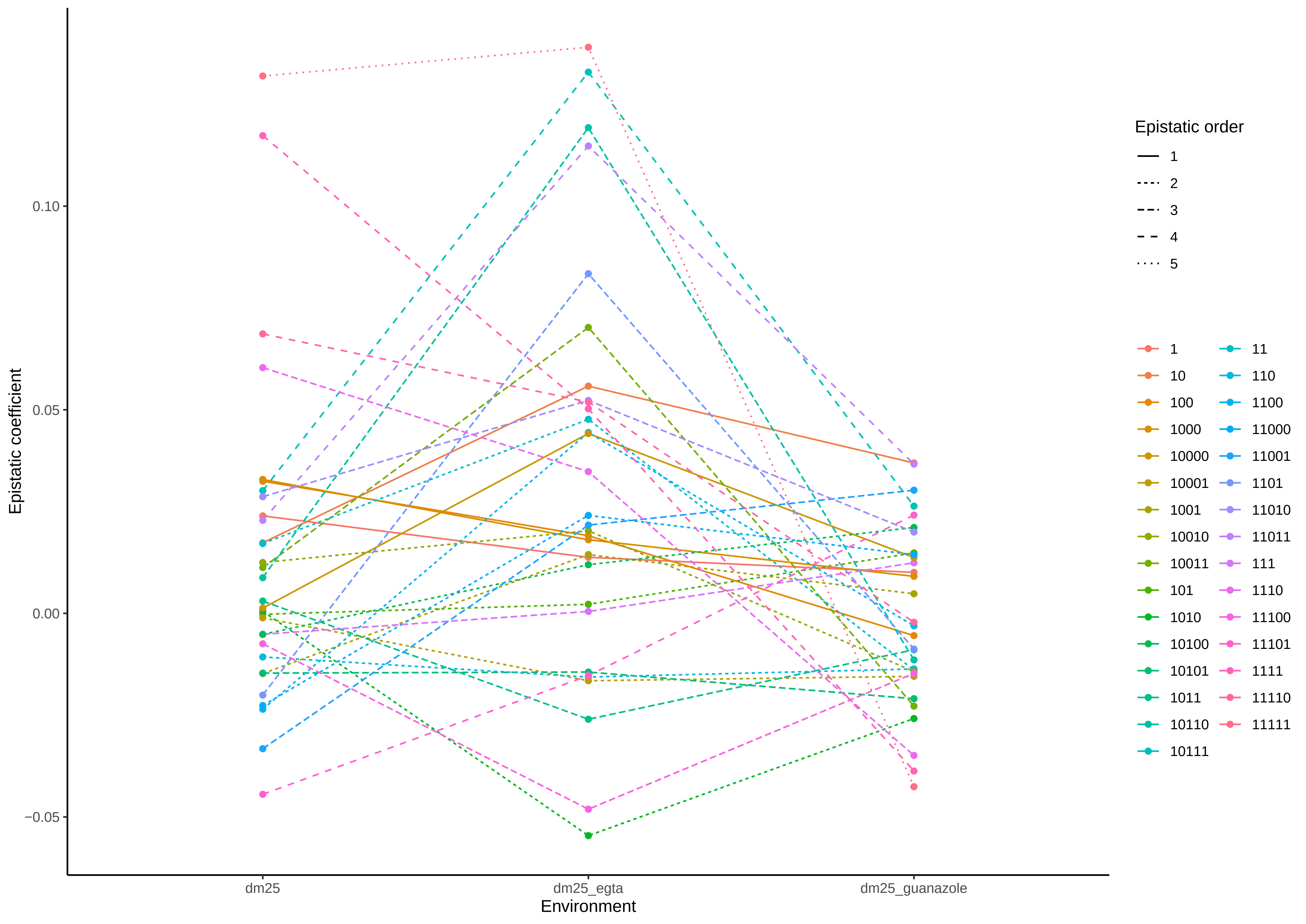

Epistatic coefficient
0.10
0.05
0.00
−0.05
dm25
dm25_egta
dm25_guanazole
Environment
Epistatic order
1
2
3
4
5
1
10
100
1000
10000
10001
1001
10010
10011
101
1010
10100
10101
1011
10110
10111
11
110
1100
11000
11001
1101
11010
11011
111
1110
11100
11101
1111
11110
11111
