# Supplementary material for: Deconstructing empirical fitness seascapes across scales of granularity
Source: G3 (Bethesda). 2026 Jun 26;16(9):jkag166. doi: 10.1093/g3journal/jkag166 (PMC13535436; doi:10.1093/g3journal/jkag166)
Supplement: Supplementary file 1 [file jkag166_supplementary_data.zip › Supplementary Material/data_code/walsh_hadamard/mira2015/murn_mira2015_20251203.pdf]

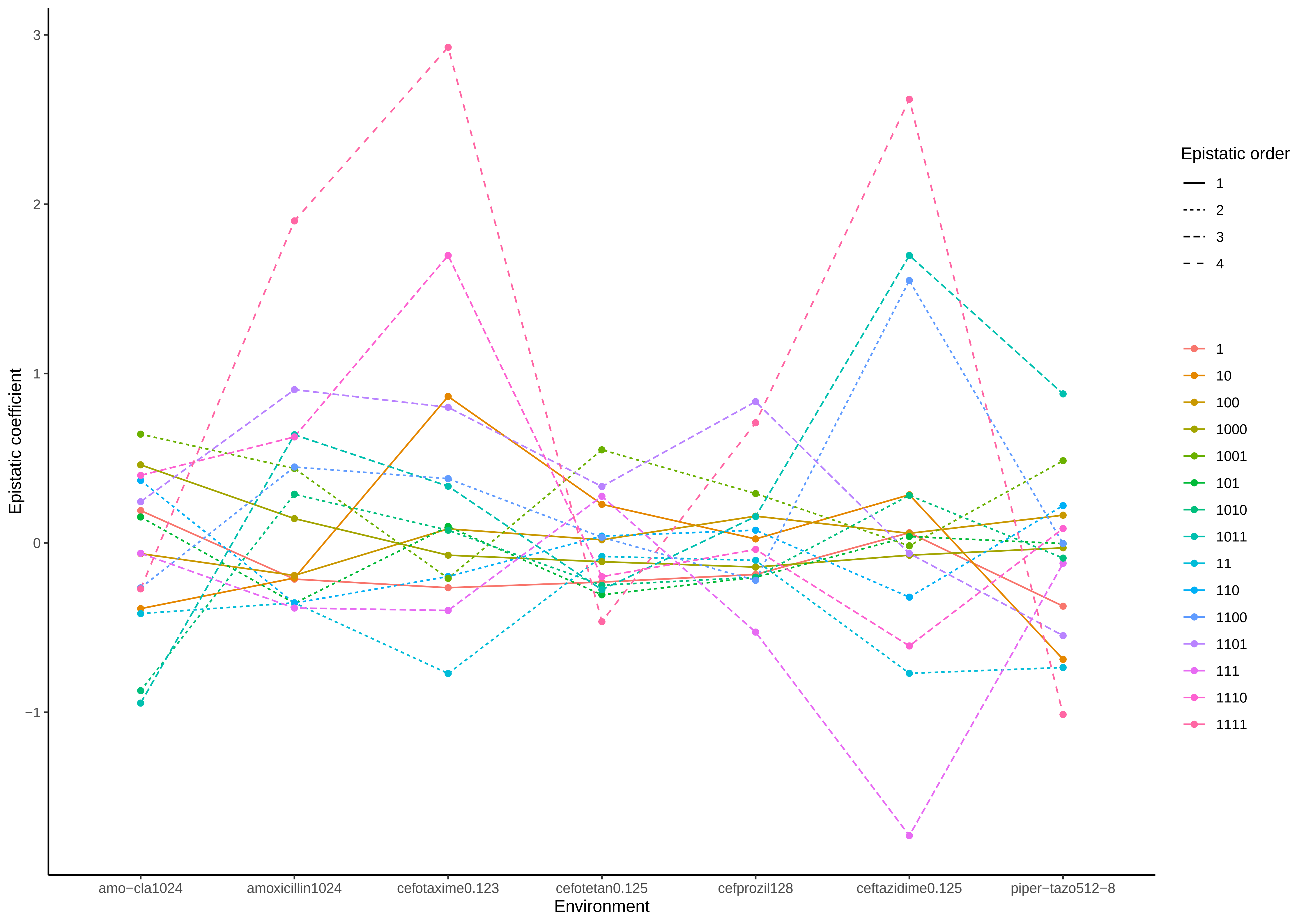

Epistatic coefficient
3
2
1
0
−1
amo−cla1024
amoxicillin1024
cefotaxime0.123
cefotetan0.125
cefprozil128
ceftazidime0.125
piper−tazo512−8
Environment
Epistatic order
1
2
3
4
1
10
100
1000
1001
101
1010
1011
11
110
1100
1101
111
1110
1111
