# Supplementary material for: Deconstructing empirical fitness seascapes across scales of granularity
Source: G3 (Bethesda). 2026 Jun 26;16(9):jkag166. doi: 10.1093/g3journal/jkag166 (PMC13535436; doi:10.1093/g3journal/jkag166)
Supplement: Supplementary file 1 [file jkag166_supplementary_data.zip › Supplementary Material/data_code/walsh_hadamard/kosterlitz2023/murn_sum_kosterlitz2023_20251205.pdf]

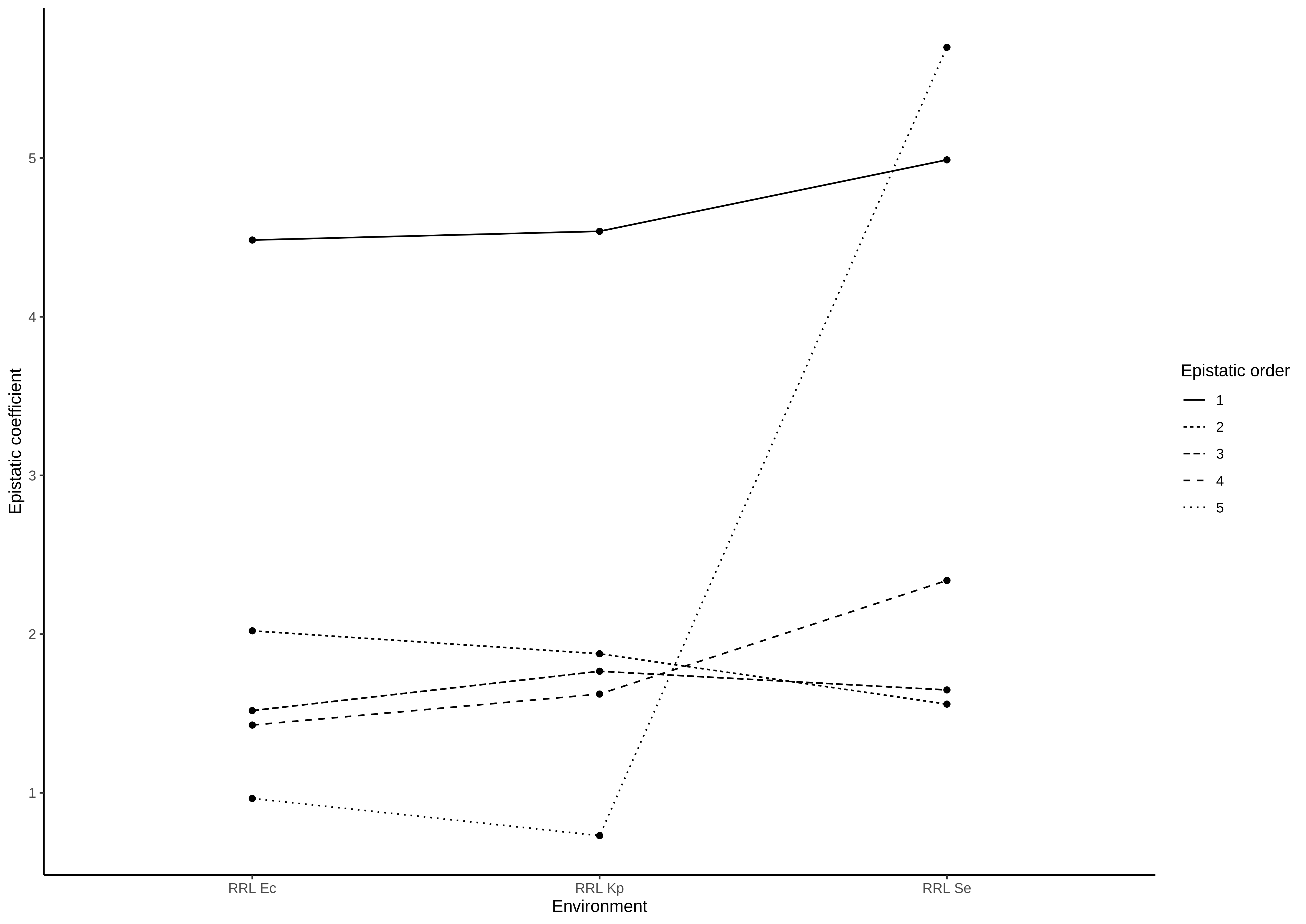

Epistatic coefficient
5
4
3
2
1
RRL Ec
RRL Kp
RRL Se
Environment
Epistatic order
1
2
3
4
5
