# Supplementary material for: Deconstructing empirical fitness seascapes across scales of granularity
Source: G3 (Bethesda). 2026 Jun 26;16(9):jkag166. doi: 10.1093/g3journal/jkag166 (PMC13535436; doi:10.1093/g3journal/jkag166)
Supplement: Supplementary file 1 [file jkag166_supplementary_data.zip › Supplementary Material/data_code/different_envts_rankorders/kosterlitz2023/kendall_kosterlitz2023_20250520.pdf]

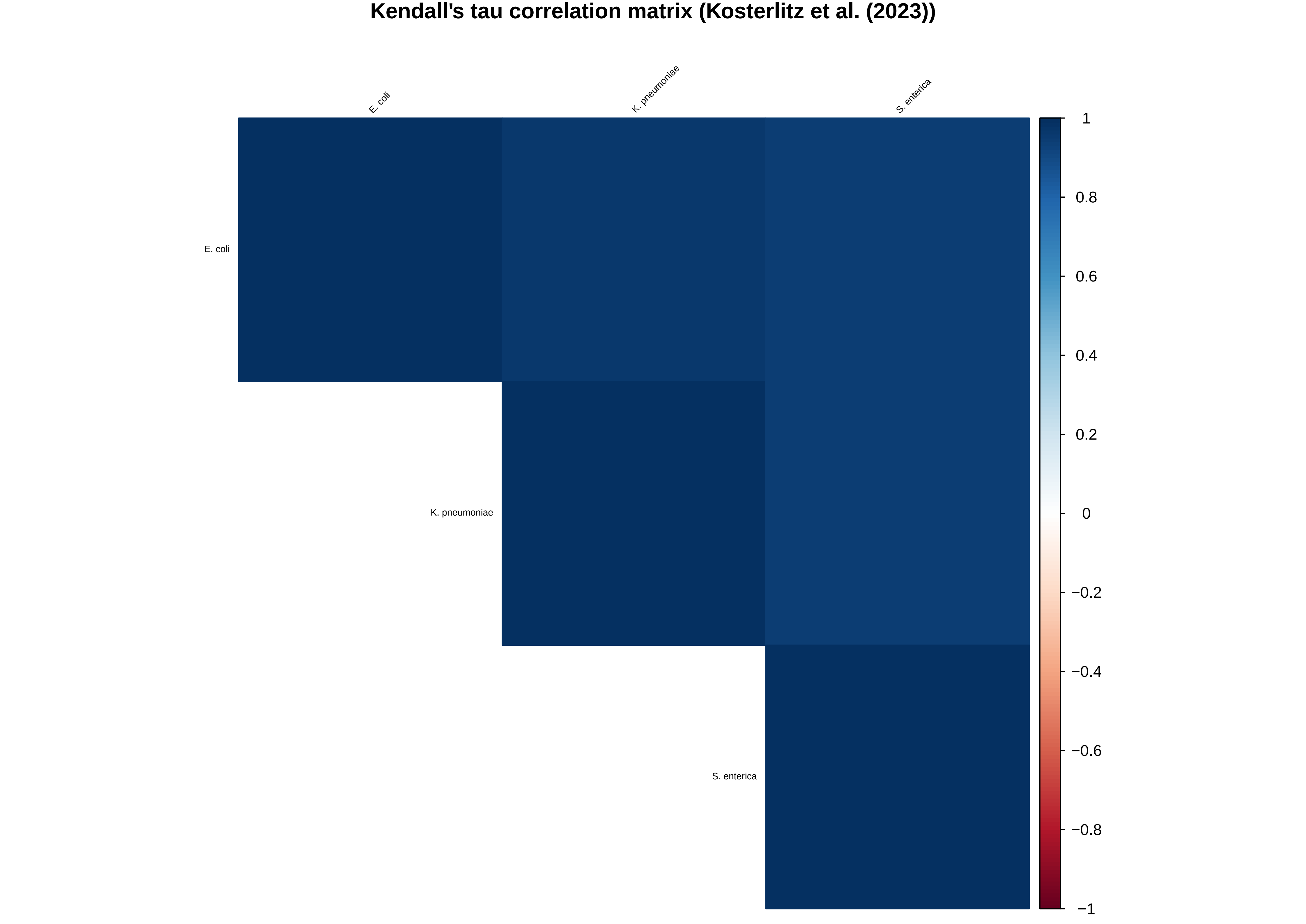
Kendall's tau correlation matrix (Kosterlitz et al. (2023))
E. coli
K. pneumoniae
S. enterica
E. coli
K. pneumoniae
S. enterica
1
0.8
0.6
0.4
0.2
0
−0.2
−0.4
−0.6
−0.8
−1
