# Supplementary material for: Deconstructing empirical fitness seascapes across scales of granularity
Source: G3 (Bethesda). 2026 Jun 26;16(9):jkag166. doi: 10.1093/g3journal/jkag166 (PMC13535436; doi:10.1093/g3journal/jkag166)

Kendall's tau correlation matrix (Guerrero et al. (2019) IC50)

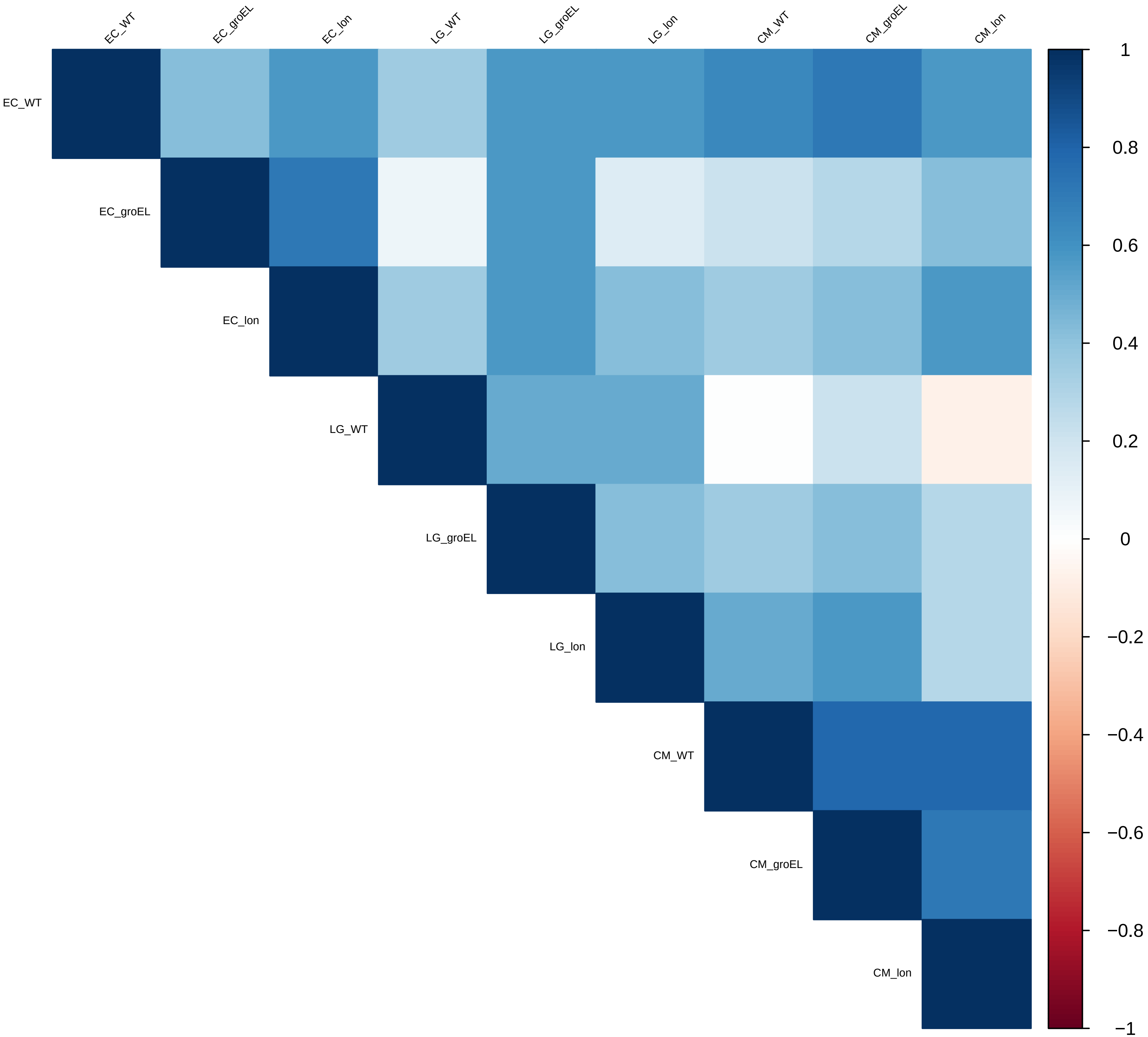

Supplement: Supplementary file 1 [file jkag166_supplementary_data.zip › Supplementary Material/data_code/different_envts_rankorders/guerrero2019/corrmatrix_guerrero_ic50_20250523.pdf]
